# The Right-Skewed Distribution of Fine-Root Size in Three Temperate Forests in Northeastern China

**DOI:** 10.3389/fpls.2021.772463

**Published:** 2022-01-07

**Authors:** Cunguo Wang, Ivano Brunner, Junni Wang, Wei Guo, Zhenzhen Geng, Xiuyun Yang, Zhijie Chen, Shijie Han, Mai-He Li

**Affiliations:** ^1^Key Laboratory of Geographical Processes and Ecological Security in Changbai Mountains, Ministry of Education, School of Geographical Sciences, Northeast Normal University, Changchun, China; ^2^Swiss Federal Institute for Forest, Snow and Landscape Research WSL, Birmensdorf, Switzerland; ^3^College of Agronomy, Shenyang Agricultural University, Shenyang, China; ^4^College of Forestry, Shanxi Agricultural University, Taigu, China; ^5^International Joint Research Laboratory for Global Change Ecology, School of Life Sciences, Henan University, Kaifeng, China

**Keywords:** branching pattern, fine-root size, first-order fine-root, inequality, root number, trade-off

## Abstract

Trees can build fine-root systems with high variation in root size (e.g., fine-root diameter) and root number (e.g., branching pattern) to optimize belowground resource acquisition in forest ecosystems. Compared with leaves, which are visible above ground, information about the distribution and inequality of fine-root size and about key associations between fine-root size and number is still limited. We collected 27,573 first-order fine-roots growing out of 3,848 second-order fine-roots, covering 51 tree species in three temperate forests (Changbai Mountain, CBS; Xianrendong, XRD; and Maoershan, MES) in Northeastern China. We investigated the distribution and inequality of fine-root length, diameter and area (fine-root size), and their trade-off with fine-root branching intensity and ratio (fine-root number). Our results showed a strong right-skewed distribution in first-order fine-root size across various tree species. Unimodal frequency distributions were observed in all three of the sampled forests for first-order fine-root length and area and in CBS and XRD for first-order fine-root diameter, whereas a marked bimodal frequency distribution of first-order fine-root diameter appeared in MES. Moreover, XRD had the highest and MES had the lowest inequality values (Gini coefficients) in first-order fine-root diameter. First-order fine-root size showed a consistently linear decline with increasing root number. Our findings suggest a common right-skewed distribution with unimodality or bimodality of fine-root size and a generalized trade-off between fine-root size and number across the temperate tree species. Our results will greatly improve our thorough understanding of the belowground resource acquisition strategies of temperate trees and forests.

## Introduction

Fine roots play a critical role in resource absorption because they are metabolic hotspots associated with symbiotic mycorrhizal fungi (Zadworny and Eissenstat, 2011; McCormack et al., 2020; Kong et al., 2021). Their size (e.g., the diameter of first-order roots) profoundly influences a variety of eco-physiological processes such as lifespan, mortality and decomposition (Eissenstat, 1992; Pregitzer et al., 2002). Many previous studies have focused on the natural variations in fine-root size and its potential ecological implications on various scales (Comas and Eissenstat, 2009; Kong et al., 2014; Freschet et al., 2017). At the species level, fine-root diameter and length vary from one to two orders of magnitude across tropical and temperate tree species, with larger roots having a thicker cortex and larger stele diameter (Gu et al., 2014; Kong et al., 2014). For a given species, the average diameter and length of an individual fine root increase steadily with root order, but the capacity of resource acquisition significantly decreases (Pregitzer et al., 2002; Guo et al., 2008). Within a root order, roots vary in diameter and length, leading to variation in absorptive capacities for water and nutrients (Polverigiani et al., 2011; Zadworny and Eissenstat, 2011). Born as first-order roots, fibrous roots with a smaller diameter and shorter length are the principal roots for water and nutrient absorption, while pioneer roots with a larger diameter and longer length are built, as the main exploratory roots, to live longer at the expense of absorptive capacity (Zadworny and Eissenstat, 2011). Plants do not put all their “eggs in one basket”, in that large fine roots co-exist with small fine roots (Forde, 2009). Accordingly, variation in the length and diameter of fine roots within a root system can be considered a strategy to reduce ecological niche overlap (e.g., root lifespan overlap) and competition between individual roots, resulting in the exploration of a greater volume of soil (Pagès et al., 1993; Pregitzer et al., 2002).

There is a tremendous amount of evidence that frequency distributions of individual plant height, seed size and leaf size are characteristically non-normal and skewed to the right (Bendel et al., 1989; Moles et al., 2007; Wang et al., 2019). The right-skewed distributions of plant size at different taxonomical levels indicate that small sizes were favored (Dombroskie and Aarssen, 2010). However, to date, the distribution characteristics of fine-root size have not been investigated in detail for forest ecosystems (Wang et al., 2017). Most temperate habitats for trees have environmental conditions where adaptation is conferred through physiological optimization associated directly with many smaller roots and few large ones (Ma et al., 2018). Thin roots have lower construction costs and are more plastic in terms of growth proliferation for resource acquisition than thick roots (Eissenstat, 1991, 1992). Thus, it is necessary to explicitly examine the frequency distribution of fine-root size to improve our ability to predict how trees will respond to a changing climate, and therefore altered resource availability, at the individual root level (Eissenstat and Achor, 1999; Wang et al., 2017). For example, in our previous study we reported that not only the mean but also the variation and the size distribution of the fine roots of *Fraxinus mandschurica* respond to changes in soil nitrogen and water availability (Wang et al., 2017). Furthermore, some plant species show a tendency of bimodal distribution in fine-root diameter (Boot and Mensink, 1990; Eissenstat, 1991; Bouma et al., 2001; Anderson et al., 2007), whereas others follow a unimodal distribution (Scanlan and Hinz, 2010; Hu et al., 2015). In conclusion, coupling the metrics of root functional characteristics with the frequency distribution of fine-root size is considered a powerful approach in assessing water and nutrient uptake behavior of the entire root system and its interaction with the soil environment (Scanlan and Hinz, 2010; Hu et al., 2015).

Plants are certainly modular organisms, with recognized capabilities to regulate the size and number of organs at the module scale (Kroon et al., 2005). If a module or structure is relatively small, plants will generally have the capacity to produce more of them (Westoby and Wright, 2003; Deng et al., 2008; Wang et al., 2019). For instance, tree species that produce smaller leaves generally build proportionately more leaves per unit size of the supporting shoot vegetative tissue (i.e., they have a higher leafing intensity), which confirms the generality of the size/number trade-off relationship, regardless of leaf form, leaf habit and habitat (Sun et al., 2006; Kleiman and Aarssen, 2007). As a central component of life-history theory, the fundamental size/number trade-off can be applied to the interpretation of fine-root size variation (Moles et al., 2007). At a specific belowground carbon allocation, trees are expected to produce a highly branched root system capable of rapid and extensive proliferation into resource-rich patches with numerous small roots (Berntson, 1994; Hodge, 2004; Chen et al., 2013). Conversely, a root system with a low level of branching has a higher exploitation potential and the overlap among depletion zones is lower for the few neighboring large roots, which typically have longer life spans and longer nutrient uptake times (Comas and Eissenstat, 2009; Liese et al., 2017). Therefore, fine-root size variation may be mainly constrained to small sizes, not because of the direct adaptability of small roots, but as a necessary trade-off consequence of selection in favor of high root branching (Ding et al., 2020; McCormack et al., 2020).

The temperate mixed forests in Northeastern China account for 35.3 and 34.5% of the national total forested land and standing tree volume, respectively (Wang, 2006). These forests function as a significant carbon sink in the national and global carbon budget (Fang et al., 2001). As the main resource absorption organs, fine roots have a considerable influence on a series of ecological processes, such as net primary production and soil formation in forest ecosystems, and their contributions to forest carbon and nutrient cycling are generally largely mediated by their functional traits, such as fine-root diameter (Freschet et al., 2017, 2021; Ma et al., 2018). However, relatively little is known about the fine-root systems in these large/important forests. In the present study, therefore, we investigated the size characteristics of fine roots in this forested area, along with their frequency distribution, inequality and associations with the number of fine roots. We focused on the first- and second-order roots, due to their important roles in generating variation among root individuals to cope with the instability of forest soil content of nitrogen and water (Pregitzer et al., 2002; Chen et al., 2013; Kong et al., 2014; Wang et al., 2017). Our first objective was to determine whether there is a consistent frequency distribution and inequality or variability in fine-root size at the root individual level within/across temperate tree species. We hypothesized that the diameter, length and area of fine-root individuals would show right-skewed distributions across different tree species and forest ecosystems (asymmetry distribution hypothesis). Next, we aimed to determine whether there is a conspicuous fine-root size/number trade-off below ground, similar to the widely established leaf size/number trade-off above ground. We hypothesized that trees in temperate forests of Northeastern China either build relatively thick fine roots but less branched fine-root systems (small root number) or produce thinner fine roots but more highly branched fine-root systems (large root number; size-number hypothesis).

## Materials and Methods

### Study Site and Root Collection

For the present study, three temperate natural mixed conifer–broadleaf forests were selected in Northeastern China: Changbai Mountain (CBS), Xianrendong (XRD) and Maoershan (MES) (Table 1). In August 2018, we sampled a total of 51 typical species (19 in XRD, 17 in CBS and 15 in MES), which spanned a wide range of fine-root size (Supplementary Table 1). At least three mature trees were chosen for each species in each forest ecosystem. Roots at 0–20 cm soil depth were carefully excavated near the base of the selected trees (Guo et al., 2008; Kong et al., 2014). Root branches with intact terminal branch orders were cut, transported in plastic bags in a cooler to the laboratory, and frozen at −20°C until subsequent morphological and chemical analyses.

**TABLE 1:** Summary of the sampling sites in the Changbai Mountain (CBS), Xianrendong (XRD) and Maoershan (MES) forests investigated in this study.

	CBS (Jilin province)	MES (Heilongjiang province)	XRD (Liaoning province)
Location	42°24′N, 127°47′E	45°21′N, 127°30′E	39°54′N, 122°53′E
Climate	Temperate, continental	Temperate, continental monsoon	Humid, warm temperate monsoon
Elevation	738 m a.s.l.	360 m a.s.l.	140 m a.s.l.
MAT	3.5°C	2.8°C	8.7°C
MAP	740 mm	723 mm	1000 mm
Soil	Eutric Cambisol with high organic matter content	Hap-Boric Luvisol with high organic matter content	Haplic Alisol with medium organic matter content

*MAT, mean annual temperature; MAP, mean annual precipitation.*

### Measurements of Fine-Root Traits and Estimations of Inequality in Fine-Root Size

In the laboratory, the sampled roots were classified into first- and second-order fine roots. Most distal roots were named first-order fine roots, while roots that contained only first-order fine roots were named second-order fine roots (Pregitzer et al., 2002). Here, the term “fine-root size” was defined as the measured values of root length, diameter and area for individual roots. We used branching intensity and ratio as a measure of root number. Branching intensity was defined as the number of first-order roots per centimeter of second-order roots, and branching ratio was defined as the number of first-order roots per second order root (Comas and Eissenstat, 2009; Kong et al., 2014). Branching intensity and ratio were measured for at least 15 second-order roots per individual tree. The collected roots were scanned with an Epson Expression 10000XL scanner (Seiko Epson Corporation, Japan). Measurements were made on the computer screen by mouse clicking on the displayed images using the measuring tools (length of a straight line and a segmented line) provided by ImageJ 1.47 software (National Institutes of Health, United States). Subsamples of fine roots from each species were cleaned and oven-dried at 60°C for 24 h and ground to a fine powder using a ball mill. Carbon and nitrogen concentrations of fine roots were determined with an elemental analyzer (Vario EL Cube; Elementar, Hanau, Germany).

### Statistical Analysis

All analyses were run in R version 4.0.3 (R Core Development Team, 2020). We used the Kruskal-Wallis test (*kruskal.test* function in the “stats” R package) and Kolmogorov-Smirnov test (*ks.test* function in the “stats” R package) to examine differences in fine-root length, diameter and area and their frequency distributions among the three sampling sites. *Post hoc* tests were used for mean values of fine-root traits using Fisher’s least significant difference criterion to explore differences among the three sampling sites when the Kruskal-Wallis chi-squared value was statistically significant (*p* < 0.05). Kernel density estimates were calculated by weighing the distance of all the data points in each specific location along the distribution (*geom_density* function in the “ggplot2” R package). The coefficient of variation (CV) and the Gini coefficient (G) (a statistic based on sums of absolute deviations of all observations) have both previously been used to quantify relative size inequality measures of plant traits and populations (He et al., 2005; Zhang and Chen, 2015; Rasmussen and Weiner, 2017). However, the coefficient of variation is more sensitive (less robust) to observations in the right-hand tail of the distribution (Bendel et al., 1989). The Gini coefficient (ranging from zero to one) is a single value that describes a specific degree of evenness, measuring the normalized area between a given Lorenz curve and the perfect evenness line (Weiner and Solbrig, 1984). As an indicator based on Lorenz curves, the Gini coefficient can deepen our insight into the overall degree of size inequality associated with fine-root traits and the relationships between fine-root inequality and distribution pattern (He et al., 2005; Wang et al., 2017). We calculated Gini coefficients with the *Lc* function in the “ineq” R package and plotted Lorenz curves with the *ggplot* function in the “gglorenz” R package with the blessing of “ggplot2” R package (Buckley and Damgaard, 2012; Chen and Cortina, 2020). We used phylogenetically controlled mixed-effects kinship models (*lmekin* function in the “coxme” R package) with random effects of tree species to explore the relationships between fine-root size (root length, diameter and area) and fine-root number (branching intensity and branching ratio). Fine-root trait values were log-transformed before analyses. Moreover, a simple correlation analysis (*ggcorr* function in the “GGally” R package) with the Spearman method and a principal component analysis using the spectral decomposition approach (*princomp* function in the “stats” R package) with standardization data were employed to assess the correlations between the fine-root size traits and their inequality. Results from the principal component analysis were visualized graphically using *fviz_pca_biplot* function in the “factoextra” R package.

## Results

### Distribution and Inequality in Fine-Root Size

**FIGURE 1:**
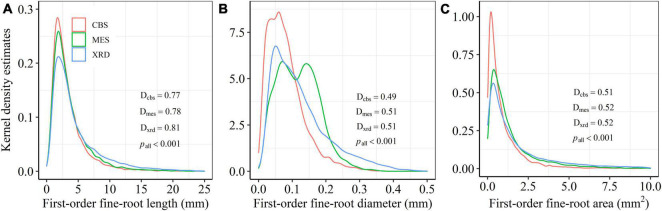
Kernel density estimates of first-order fine-root length **(A)**, diameter **(B)** and area **(C)** for Changbai Mountain (CBS), Maoershan (MES), and Xianrendong (XRD) forests. D and *p*-values from Kolmogorov-Smirnov tests are shown for each forest (D_*cbs*_, D_*mes*_, and D_*xrd*_).

In total, 27,573 first-order fine roots from 3848 second-order fine roots across 51 tree species from the three temperate forest ecosystems were collected to assess the distribution and inequality of fine-root size (Supplementary Tables 1, 2). The frequency distributions from the Kolmogorov-Smirnov tests showed marked right-asymmetry for first-order fine-root length, diameter and area for the three forest ecosystems in Northeastern China (Figure 1). The positive skewness indicated that the distributions of first- and second-order fine-root size were right-skewed for almost all species, with a systematic right-hand tail of the distributions (Supplementary Table 2 and Supplementary Figures 1–3). The three forest ecosystems showed a similar frequency distribution pattern in first-order fine-root length, with a unimodal value of about 1.80 mm (Figure 1A). A markedly bimodal frequency distribution of first-order fine-root diameter was observed in MES only, with one peak at about 0.07 mm and a second peak at about 0.14 mm (Figure 1B). The similar unimodal frequency distribution patterns for first-order fine-root area in the three sites were leptokurtic, with the greatest proportion of small first-order fine-roots appearing in CBS (Figure 1C).

**FIGURE 2:**
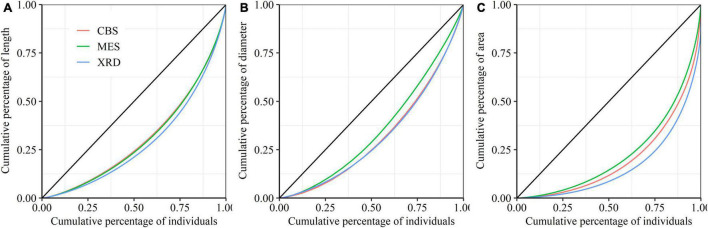
Lorenz curves for the length **(A)**, diameter **(B)** and area **(C)** of first-order fine roots from Changbai Mountain (CBS), Maoershan (MES) and Xianrendong (XRD) forests.

There were significant differences in the average length, diameter and area of first-order fine roots among the three forest ecosystems, with the largest roots appearing in XRD and the smallest roots appearing in CBS (Table 2). Fine roots in MES exhibited a higher branching intensity and ratio, while fine roots in XRD showed the lowest branching intensity and ratio (Table 2). Fine roots of the three forest ecosystems had a similar average coefficient of variation (CV) and Gini coefficient (G) of first-order fine-root length and area. However, significant differences in the Gini coefficients of first-order root diameter were detected, with the highest and lowest values being observed in XRD and MES, respectively (Table 2 and Figure 2). Furthermore, large variations in first- and second-order fine-root size were found both within and among species in the three forest ecosystems (Supplementary Tables 1, 2). At the species level, *Phellodendron amurense* produced the largest first- and second-order roots but had smaller variation and inequality in fine-root size (lower CV and G) and lower branching intensity and ratio within each forest ecosystem (Supplementary Tables 1, 2 and Supplementary Figures 4–6). *Sorbaria sorbifolia*, *Corylus mandshurica* and *Quercus mongolica* had the smaller first- and second-order fine roots but had higher variation and inequality in fine-root size (higher CV and G) (Supplementary Tables 1, 2 and Supplementary Figures 4–6). *Ulmus davidiana*, *C. mandshurica* and *Ulmus laciniata* had the highest branching intensity and ratio across the three forest ecosystems (Supplementary Table 3).

**TABLE 2:** Differences in the size and number, and their inequality/variation, of the first-order fine roots collected from Changbai Mountain (CBS), Xianrendong (XRD) and Maoershan (MES) forests.

	No	Mean ± SE	Median	Maximum	Minimum	Skewness	Kurtosis	CV	Gini
**Changbai mountain forest (CBS**)									
FL	9218	3.43 ± 0.03*^c^*	2.63	52.6	0.01	3.27	22.4	0.83	0.30 ± 0.01*^a^*
FD	9218	0.08 ± 0.01*^c^*	0.07	0.60	0.01	1.51	3.86	0.75	0.22 ± 0.01*^b^*
FA	9218	1.10 ± 0.02*^c^*	0.56	42.1	0.01	6.01	58.2	1.71	0.42 ± 0.02*^a^*
BR	1274	7.24 ± 0.12*^b^*	6.00	30.0	1.00	1.40	2.41	0.57	
BI	1274	4.73 ± 0.09*^b^*	4.04	27.0	0.55	2.15	8.15	0.65	
**Maoershan forest (MES)**									
FL	9203	3.73 ± 0.03*^b^*	2.99	26.9	0.09	2.29	7.56	0.80	0.30 ± 0.01*^a^*
FD	9203	0.12 ± 0.01*^b^*	0.12	0.71	0.01	0.63	1.26	0.50	0.15 ± 0.01*^c^*
FA	9203	1.61 ± 0.02*^b^*	0.91	25.1	0.01	3.70	19.7	1.31	0.35 ± 0.01*^a^*
BR	1156	7.97 ± 0.13*^a^*	7.00	33.0	1.00	1.40	2.56	0.55	
BI	1149	4.98 ± 0.09*^a^*	4.43	34.8	0.46	2.57	18.0	0.61	
**Xianrendong forest (XRD)**									
FL	9152	4.65 ± 0.04*^a^*	4.17	49.6	0.03	2.42	9.96	0.90	0.29 ± 0.01*^a^*
FD	9152	0.13 ± 0.01*^a^*	0.10	1.03	0.01	1.49	4.15	0.69	0.26 ± 0.01*^a^*
FA	9152	2.34 ± 0.04*^a^*	1.09	65.1	0.01	4.23	32.5	1.52	0.46 ± 0.02*^a^*
BR	1425	6.42 ± 0.10*^c^*	5.00	31.0	1.00	1.54	3.60	0.58	
BI	1425	4.01 ± 0.08*^c^*	3.19	30.8	0.29	2.15	8.13	0.79	

*Different letters within a column indicate statistical significance at p < 0.05. FL, first-order fine-root length (mm); FD, first-order fine-root diameter (mm); FA, first-order fine-root area (mm^2^); BR, branching ratio; BI, branching intensity. No, number of individual fine roots; CV, coefficient of variation.*

### Correlations Among Different Fine-Root Traits

**FIGURE 3:**
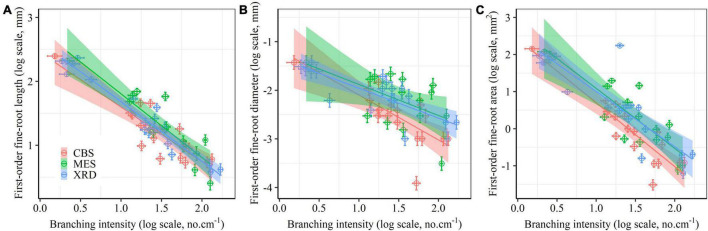
Relationships between the size [length **(A)**, diameter **(B)** and area **(C)**] of first-order fine roots and branching intensity for Changbai Mountain (CBS), Maoershan (MES) and Xianrendong (XRD) forests. Linear mixed-effects kinship model (Supplementary Table 4) fits are displayed, along with 95% confidence intervals.

**FIGURE 4:**
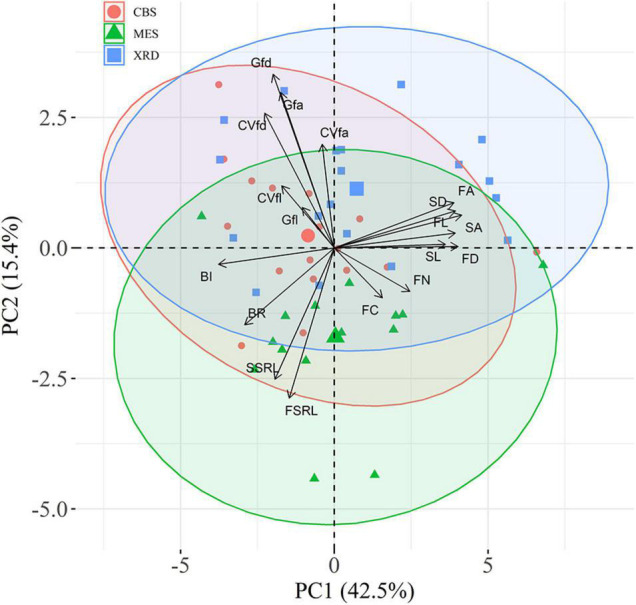
Principal component analysis (PCA) of fine-root traits for Changbai Mountain (CBS), Maoershan (MES) and Xianrendong (XRD) forests. The 95% confidence ellipses are shown. FL, first-order fine-root length; FD, first-order fine-root diameter; FA, first-order fine-root area; FSRL, specific root length of first-order fine roots; SL, second-order fine-root length; SD, second-order fine-root diameter; SA, first-order fine-root area; SSRL, specific root length of second-order fine roots; BR, branching ratio; BI, branching intensity; FC, carbon content of first-order fine roots; FN; nitrogen content of first-order fine roots; Gfl, Gini coefficient of FL; Gfd, Gini coefficient of FD; Gfa, Gini coefficient of FA; CVfl, coefficient of variation of FL; CVfd, coefficient of variation of FD; CVfa, coefficient of variation of FA.

First-order fine-root size showed a consistently linear decline with increasing branching intensity and ratio (Figure 3 and Supplementary Figure 7), although the negative relationship between fine-root diameter and branching ratio was not significant in MES (Supplementary Table 4). First-order fine-root size was strongly positively correlated with the size of second-order roots (Supplementary Figure 8). There were evidently positive correlations among root length, root diameter and root area for both root orders (Supplementary Figure 8). The first two trait axes of the principal component analysis accounted for 42.5 and 15.4% of the total variation, respectively (Figure 4). Branching intensity and ratio and fine-root size-related parameters had high scores on the first axis, while inequality and variation parameters for first-order fine-root diameter and area had high scores on the second axis (Figure 4 and Supplementary Table 5). Additionally, length inequality and variation, nitrogen and carbon content of first-order fine-roots, and specific root length of first- and second-order fine roots had relatively high scores on the third axis (Figure 4 and Supplementary Table 5). Tree species in XRD tended to have higher fine-root size inequality and variation, while those in MES tended to have higher root branching. Fine roots of tree species from CBS scattered on both axes of the principal component analysis (Figure 4).

## Discussion

### The Right-Skewed Distribution of Fine-Root Size

In support of our asymmetry distribution hypothesis, we observed noticeably right-skewed distributions of fine-root size (length, diameter and area) at the individual level, with a long tail of larger roots, for the three temperate forest ecosystems and for most of the sampled tree species within each ecosystem in Northeastern China. The preponderance of the right-skewed distribution of plant organ size (small fine-roots in this study) is considered a consequence of the left-wall effect, because the sizes of organs must be greater than zero (Jensen and Zwieniecki, 2013). Similar to leaf size, the strongly right-skewed distributions for fine-root size at the community and species level suggest that most species have experienced natural selection favoring effects of relatively small fine-roots. Stated another way, trees that invest in a small number of coarse roots and many fine roots of small diameter may be better adapted for intensive fine-root growth and high fine-root length densities (Eissenstat, 1992). However, we also noted the diverse skewness and kurtosis of fine-root size among tree species in the three forest ecosystems. For instance, fine-root diameter of *P. amurense* with the negative skewness demonstrated a left-skewed distribution pattern (i.e., many large fine-roots) in both CBS and MES. These findings, similarly to previous studies, suggest that different tree species appear to present contrasting root types (e.g., species with thinner roots *versus* species with thicker roots) to enhance nutrient acquisitions in temperate and subtropic forest ecosystems (Liu et al., 2015; Chen et al., 2016; Cheng et al., 2016). Generally, the change in fine-root size is strongly related with tree’s mycorrhizal association, such as arbuscular mycorrhizal or ectomycorrhizal (Bergmann et al., 2020). Like some tree species with thick roots in a subtropical forest (Liu et al., 2015), *P. amurense* in temperate forests producing the largest roots perhaps also relies more on arbuscular mycorrhizal fungi to forage nutrients. Therefore, the integrated consideration of the tree species composition with contrasting specific fine-root and mycorrhizal traits will improve our insights into the skewed distribution of fine-root size across various tree species.

Apart from asymmetry, we found that the unimodal distribution for fine-root size (except for root diameter in MES) was leptokurtic with a narrow peak, reflecting the occurrence of strong environmental filtering in the three temperate forests in Northeastern China. Environmental filtering plays a major role in shaping trait diversity of fine roots in forests, favoring convergence to an optimal trait value (Wang R. et al., 2018). However, a bimodal platykurtic distribution of fine-root diameter was observed in MES, as previously found for other tree and grass species (Boot and Mensink, 1990; Eissenstat, 1991; Bouma et al., 2001; Anderson et al., 2007). Bimodal trait distributions arise when there are multiple optimal trait values, suggesting the co-existence of contrasting functional strategies or the occurrence of stabilizing niche differences among interacting species (Gross et al., 2013; Le Bagousse-Pinguet et al., 2016). The co-occurrence of species with thick roots *versus* species with thin roots was observed at the species level in MES temperate forest. This fits with our above discussions on the skewed-distribution of fine-root size, implying that the maintenance of large variances in fine-root size is also strongly related to the species diversity of the forest community or ecosystem. Although there is a growing body of literature on ecological implications of fine-root diameter (Fort and Freschet, 2020; Valverde-Barrantes et al., 2020), more research on root diameter distributions is required to judge the prevalence of the bimodality observed in MES and to understand fully the functional significance of the bimodal distribution in other plant species. In conclusion, the important characteristics of the right-skewed distributions at the individual level in fine-root size traits reported here provide a basis for inferences about mechanisms of exploitation and acquisition in resources, and they avoid the loss of information associated with average metrics of root systems.

### Inequality of Fine-Root Size

Our principal component analysis indicated that the second axis associated with fine-root size inequality is likely a critical dimension of the belowground resource uptake strategy in the multidimensional space of fine-root trait variation. Plant responses to environmental changes depend not only on the mean but also on the plasticity and variation of plant traits (Wang et al., 2017; Zhang et al., 2020). Further analyses extending beyond trait means will permit us to explore original relationships among the traits of interest (Russell et al., 2014). Size inequality of individuals exists in most plant populations, and the associated inequality of plant functional traits may have substantial ecological implications (Metsaranta and Lieffers, 2008; Forde, 2009). In this study, we detected significant differences in the Gini coefficients of fine-root diameter among the three forest ecosystems, with the highest and lowest values occurring in XRD and MES, respectively. Changes in fine-root size inequality have been attributed to alterations in the mode of competition among fine-root individuals, leading to changes in the shape of the root size frequency distribution of fine-root systems (Wang et al., 2017). Some previous studies have underlined the importance of competition effects on size inequality and *vice versa* (Chu et al., 2009; Rasmussen and Weiner, 2017). Efficient water availability and higher temperature in XRD are expected to favor larger fine roots growing at relatively higher rates (Kong et al., 2014; Ma et al., 2018), to increase the asymmetric competition in resource utilization by larger fine roots. The harsher environments in MES likely lead to generate many smaller fine roots growing at relatively similar rates, promoting relatively size-symmetric competition among fine-root individuals, and therefore, resource utilization is equal or proportionate to fine-root size (Pagès et al., 1993). Consequently, the biotic factors such as competition, abiotic factors such as nutrient availability and their interactions presented potential effects on size inequality of plant community (Boot and Mensink, 1990; Chu et al., 2009). In addition, we only observed significant differences in size inequality for fine-root diameter, which was always less than that for fine-root length or area in the three forest ecosystems. Therefore, the size inequality of fine-root diameter at the individual root level seems to be a better measure of size inequality, because fine-root diameter is more stable and more directly linked to the functioning of fine-root systems than fine-root length and area (Chen et al., 2013; Kong et al., 2014).

### Trade-Off Between Fine-Root Size and Fine-Root Number

In support of our size-number hypothesis, we found that fine-root size was negatively correlated with fine-root number (branching intensity and ratio), indicating a trade-off across the investigated tree species between building fewer fine roots with a relatively large diameter and forming thinner fine roots in a more highly branched fine-root system. The results reported here may represent generalized trade-off strategies for fine-root deployment at the individual root level within temperate tree species. The fine-root size/number trade-off is in line with the various well-known compromises between size and number of other organs in plant bodies, such as leaf size/number and seed size/number trade-offs (Westoby and Wright, 2003; Milla, 2009), and with compromises between size and number of individuals at the population level (Deng et al., 2008). The leafing premium hypothesis states that high branching intensity should be rewarded in natural selection, based on the pattern that most species are small-leaved and the negative relationship between leaf size and number (Kleiman and Aarssen, 2007; Leigh et al., 2017). Just as the arrangement and location of leaves on a branch influence the capture of CO_2_ and sunlight, the position and organization of most distal roots within the root system (branching pattern) regulate their various ecological and functional aspects, such as the resource-foraging strategy and intra-branch competition (Bentley et al., 2013; Kong et al., 2014; McCormack et al., 2020). A high root branching intensity may indeed bring about selective advantages, given the preponderance of small fine-roots in most tree species (e.g., the strongly positive right-skewed fine-root size frequency distribution observed in this study). Trees can enhance resource absorption not only by producing thinner roots but also by enhancing root branching intensity to rapidly exploit resource-rich soil patches at low fertility sites (Holdaway et al., 2011; Beidler et al., 2015). The fine-root size/number trade-off is one of the fundamental adaptation strategies of plants to environmental changes, and it may therefore have particularly important implications for understanding root size evolution (López-Bucio et al., 2003; Rich and Michelle, 2013; Kong et al., 2014).

We observed that larger fine-root diameters were associated with longer length of fine roots, which appears to be a simply physical relationship (Tyree and Ewers, 1991; McCormack et al., 2020), while root diameter could also be related to other functions such as storage, aerenchyma, etc., Gu et al. (2014). Longer roots, with a greater potential to absorb resources, should be connected to roots with a larger diameter, which effectively represent larger pipes with a lower resistance to conduct those resources (Kong et al., 2014; McCormack et al., 2020). Similarly, the strong, positive correlations in fine-root diameter between first- and second-order fine roots observed across tree species and within the same tree species demonstrate that relatively large first-order fine roots tend to arise from relatively large second-order roots.

### Implications of Fine-Root Size/Number Trade-Off

From an anatomical perspective, smaller fine roots have a less suberized and less lignified hypodermis than larger fine roots, characteristics that are associated with higher construction and maintenance costs (Fitter et al., 1991; Pregitzer et al., 1993; Fort and Freschet, 2020). The lower within-root structural investment for smaller fine roots of temperate tree species allows shorter life spans/quicker turnover and greater plasticity in root growth proliferation (Eissenstat, 1991, 1992), conferring them advantages in seasonally more variable environments (such as MES in this study) in terms of temperature, moisture and nutrient supply (Chen et al., 2013). For instance, small roots with a thinner cortical thickness are likely to have less hydraulic resistance to the lateral transport of resources from the root surface to the vascular bundle (Valverde-Barrantes et al., 2020). From an ecological perspective, roots with a high branching intensity (namely small roots) are capable of rapid and extensive proliferation into resource-rich patches in heterogeneous forest soils (Hodge, 2004; Kong et al., 2014). Moreover, our result of intensive root branching also increases the specific root length of the pool of first- and second-order roots, which are presumed to have high respiration rates and high resource uptake activities (Rewald et al., 2011, 2014; McCormack et al., 2015). It has previously been demonstrated that small roots enhance small-scale foraging and nutrient absorption in the arid temperate zone (Chen et al., 2013). Fine-root clusters with more root tips and branching can accommodate greater colonization by mycorrhizal fungi, which in turn improve the nutrient and water absorption capacity of fine-root systems (Comas and Eissenstat, 2009; Ekblad et al., 2016). In addition, branching pattern differences have various effects on the ability of a fine-root system to capture relatively mobile *versus* immobile soil nutrients (Beidler et al., 2015). A large number of small fine roots are needed to acquire less mobile soil resources rapidly, improving competitive ability (Robinson et al., 1999) but leading to decreases in exploitation efficiency (Berntson, 1994). Radial resistance in fine roots is an important limitation to water and nutrient uptake; therefore, fine roots with a small diameter have a shorter path length for water and nutrient movement to the xylem than large-diameter fine roots (Purushothaman et al., 2013; Wang W. et al., 2018). As a result of selection favoring minimization of transport distances and maximization of exchange surfaces, the formation of very fine roots can be a prime strategy to increase the soil–root exchange surface at a minimal cost (Kong et al., 2017).

In our study, as fine-root length increased the proliferation of root tips decreased, which may be related to the exploitation potential, i.e., the volume of soil exploited per unit volume of fine roots (Chen et al., 2016; McCormack et al., 2020). Compared with small fine roots, large fine roots are more efficient at assimilating diffuse resources through more extensive soil exploration, because low root branching intensity reduces the overlap of depletion zones of neighboring branches and inter-root individual competition (Berntson, 1994; Beidler et al., 2015). Thus, decreased frequency of root tip proliferation events along a length of fine root may be indicative of a more exploratory growth strategy (Beidler et al., 2015). In other words, there is a clear trade-off between the exploitation efficiency and exploitation potential in fine-root systems. Small fine roots in MES will show a high exploitation efficiency (intensive) but low exploitation potential and are associated with a greater specific root length, more root branching and shorter root life span (fast absorption strategy) (Comas and Eissenstat, 2009; Liese et al., 2017). Inversely, large fine roots in XRD will exhibit a high exploitation potential (extensive) but low exploitation efficiency and are associated with lower specific root length, less root branching and longer root life span (slow absorption strategy) (Zadworny and Eissenstat, 2011; Fort and Freschet, 2020). Together with the similar functional associations identified in leaves, the intensive–extensive continuum in fine-root systems is a key feature of trees and should be considered in order to gain a thorough understanding of the belowground ecological strategies of trees, the assembly processes of forest communities, and the functioning of forest ecosystems (Reich, 2014).

## Conclusion

Based on a large dataset of 27,573 individual first-order fine roots from three temperate forest ecosystems in Northeastern China, we explored the size distribution pattern of fine roots and their relationships with fine-root number. Our results reveal the overall generality of a cross-species right-skewed distribution in fine-root size traits, and confirm the existence of a trade-off between the number of fine roots and the size of fine roots of temperate tree species. The observed frequency distributions of fine-root size traits indicate that thinner roots within fine-root systems are favored over thicker roots because of their relative benefits in terms of resource acquisition. We propose that the fine-root size/number trade-off is likely to provide fundamental insight into the variation in patterns of fine-root traits in forest ecosystems. Furthermore, the relationship has important implications regarding the contrasting resource acquisition strategies of thinner and thicker roots within fine-root systems. Coupling information on fine-root size frequency distributions with knowledge on the trade-offs between fine-root functional traits will be a powerful approach in studying the water and nutrient uptake behavior of fine-root system in forest ecosystems.

## Data Availability Statement

The raw data supporting the conclusions of this article will be made available by the authors, without undue reservation.

## Author Contributions

CW and XY conceived the ideas and designed methodology. JW, WG, and ZG collected the data. CW and SH analysed the data. IB, ZC, and M-HL led the writing of the manuscript. All authors contributed critically to the drafts and gave final approval for publication.

## Conflict of Interest

The authors declare that the research was conducted in the absence of any commercial or financial relationships that could be construed as a potential conflict of interest.

## Publisher’s Note

All claims expressed in this article are solely those of the authors and do not necessarily represent those of their affiliated organizations, or those of the publisher, the editors and the reviewers. Any product that may be evaluated in this article, or claim that may be made by its manufacturer, is not guaranteed or endorsed by the publisher.
