# Supplementary material for: The Right-Skewed Distribution of Fine-Root Size in Three Temperate Forests in Northeastern China
Source: Front Plant Sci. 2022 Jan 7;12:772463. doi: 10.3389/fpls.2021.772463 (PMC8777189; doi:10.3389/fpls.2021.772463)
Supplement: Supplementary file 1 [file Data_Sheet_1.docx]

Supplementary Material

# Supplementary Figures and Tables

## Supplementary Figures


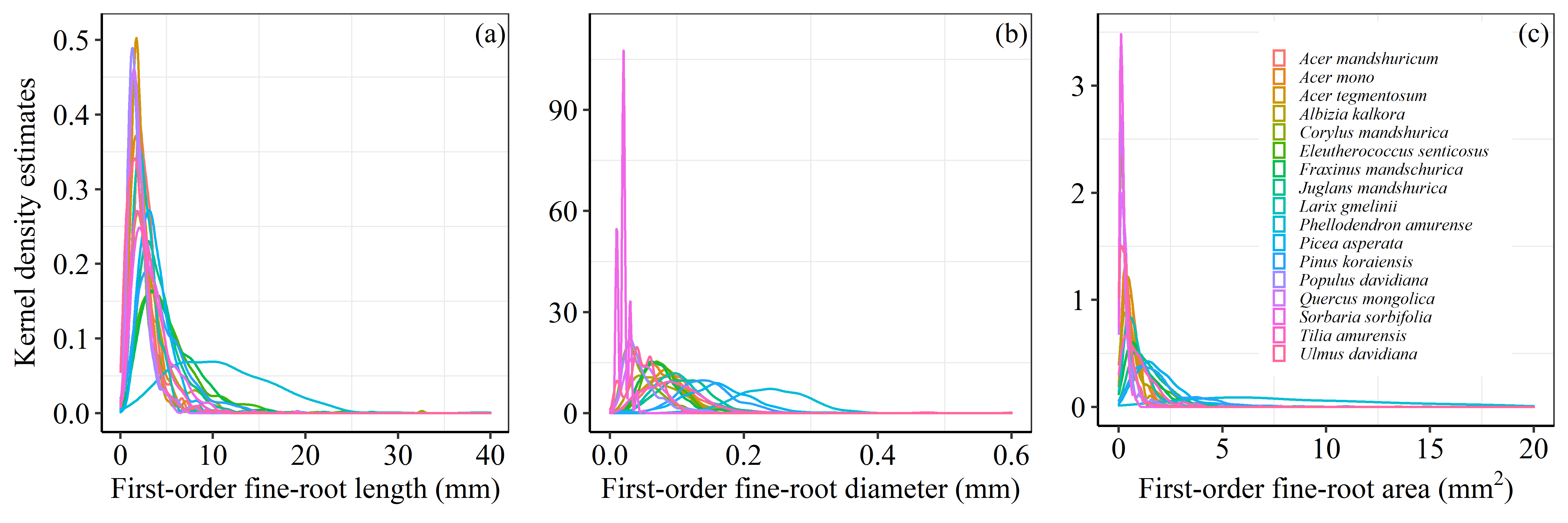


**Supplementary Figure 1.** Kernel density estimates of first-order fine-root length (a), diameter (b) and area (c) for different tree species from the Changbai Mountain forest.


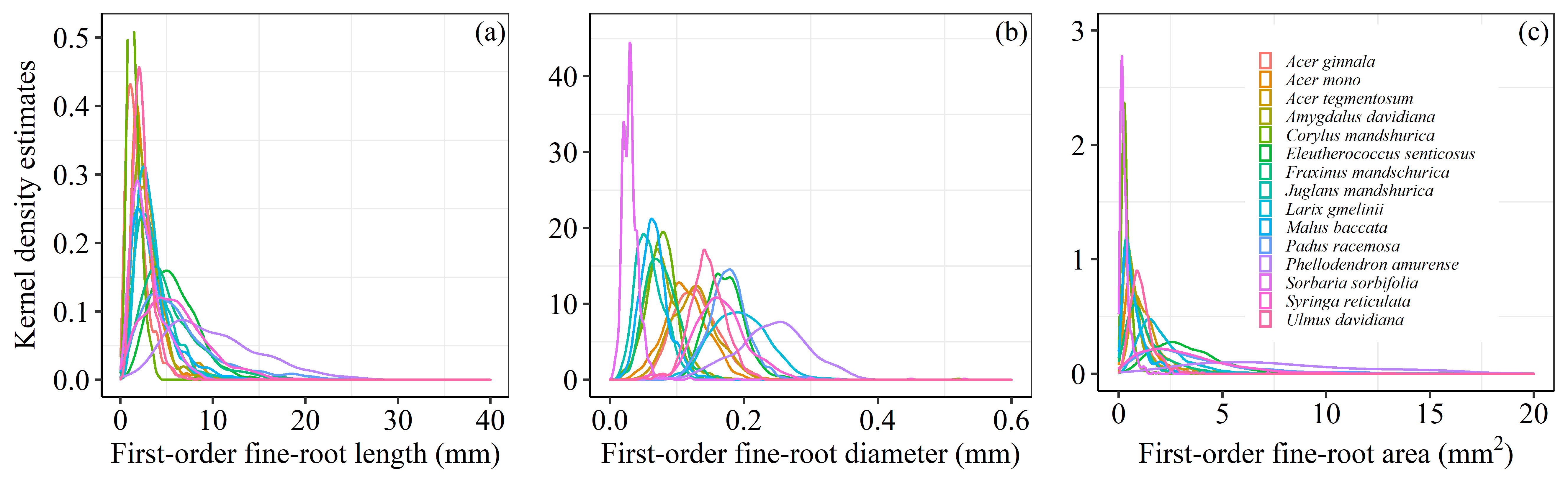


**Supplementary Figure 2.** Kernel density estimates of first-order fine-root length (a), diameter (b) and area (c) for different tree species from the Maoershan forest.


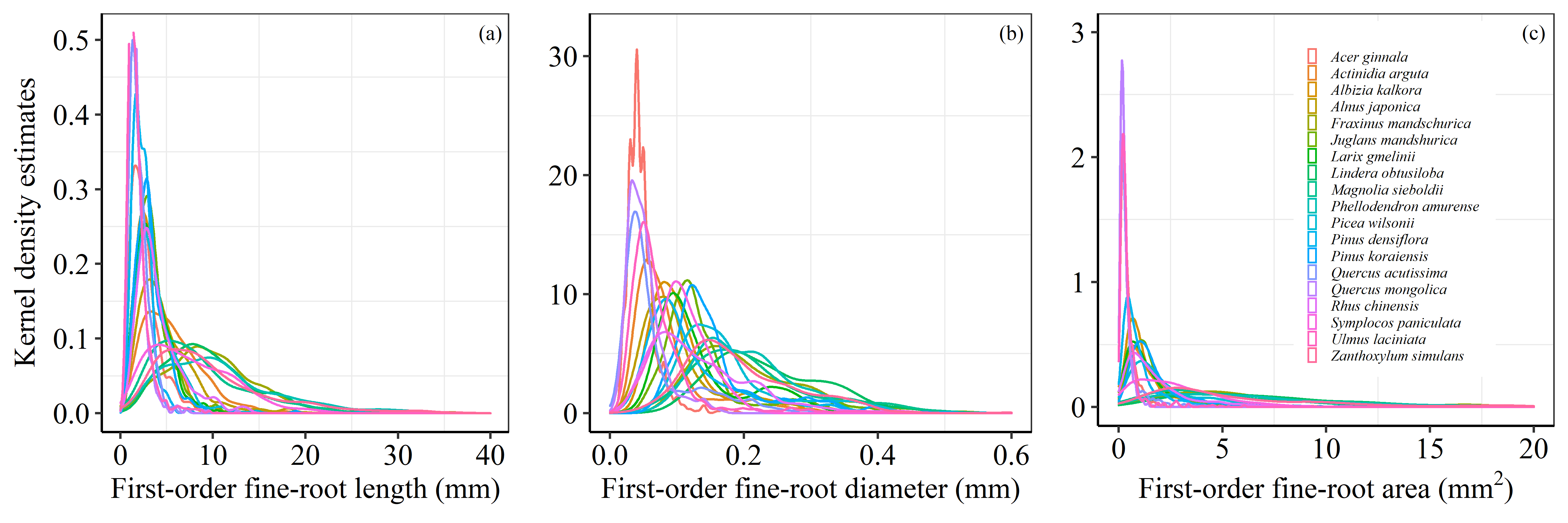


**Supplementary Figure 3.** Kernel density estimates of first-order fine-root length (a), diameter (b) and area (c) for different tree species from the Xianrendong forest.


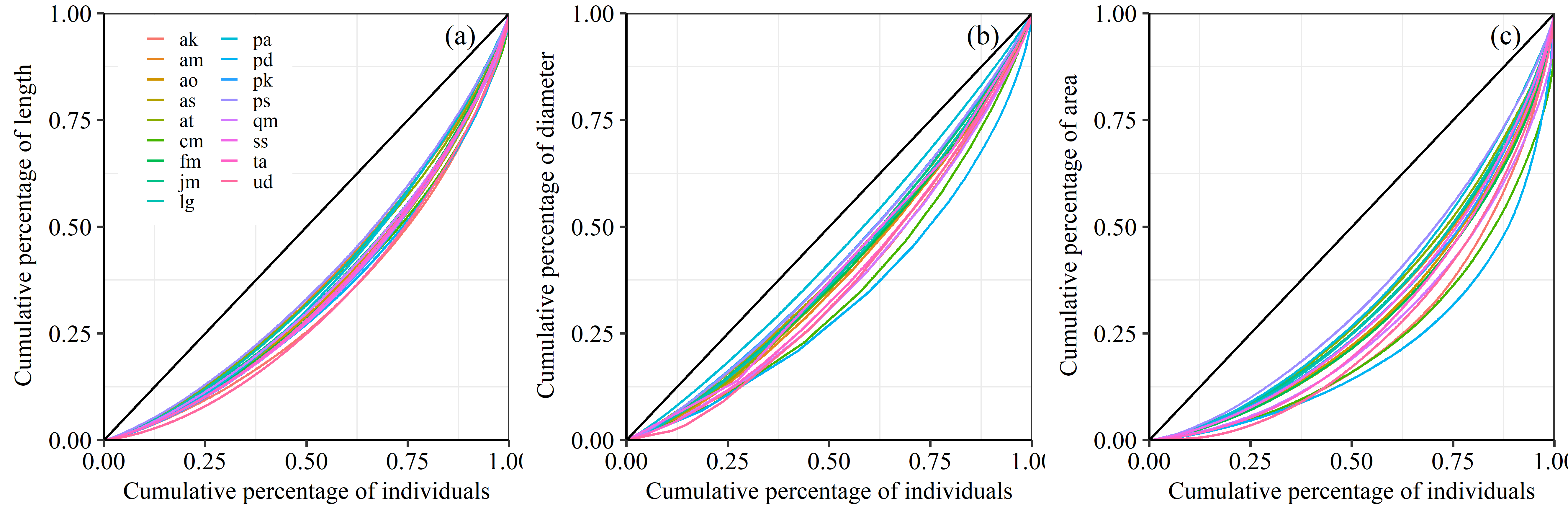


**Supplementary Figure 4.** Lorenz curves of the length (a), diameter (b) and area (c) of first-order fine roots across tree species from the Changbai Mountain forest. Abbreviations for tree species are given in Table S1.


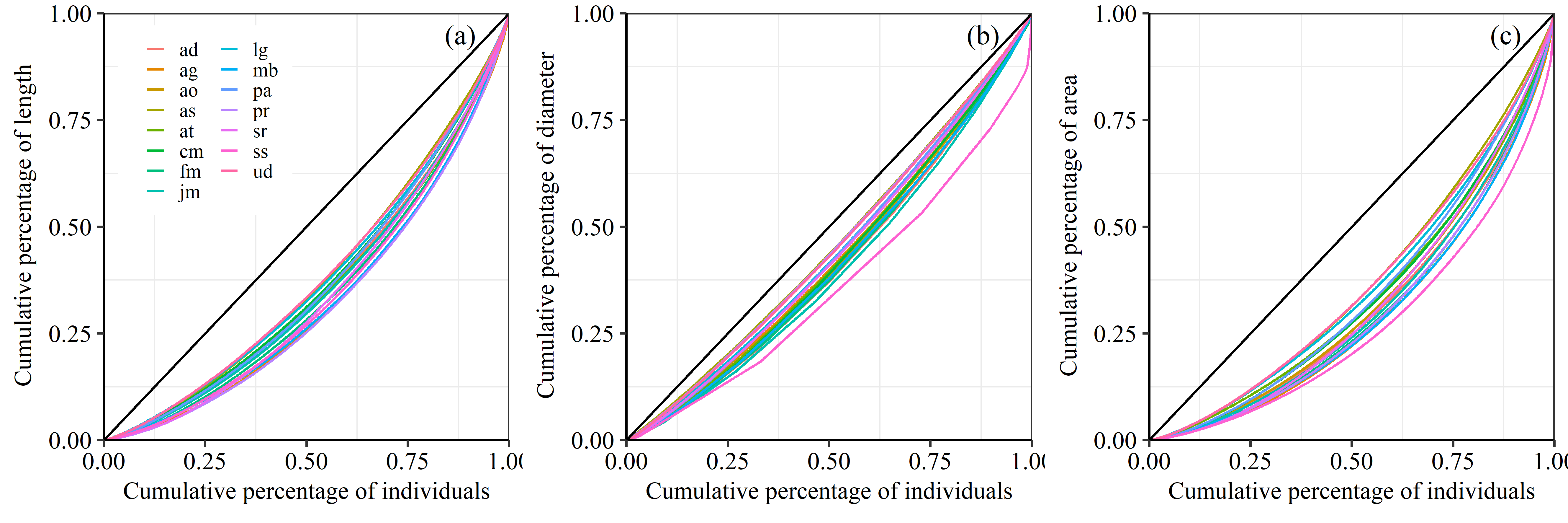


**Supplementary Figure 5.** Lorenz curves of the length (a), diameter (b) and area (c) of first-order fine roots across tree species from the Maoershan forest. Abbreviations for tree species are given in Table S1.


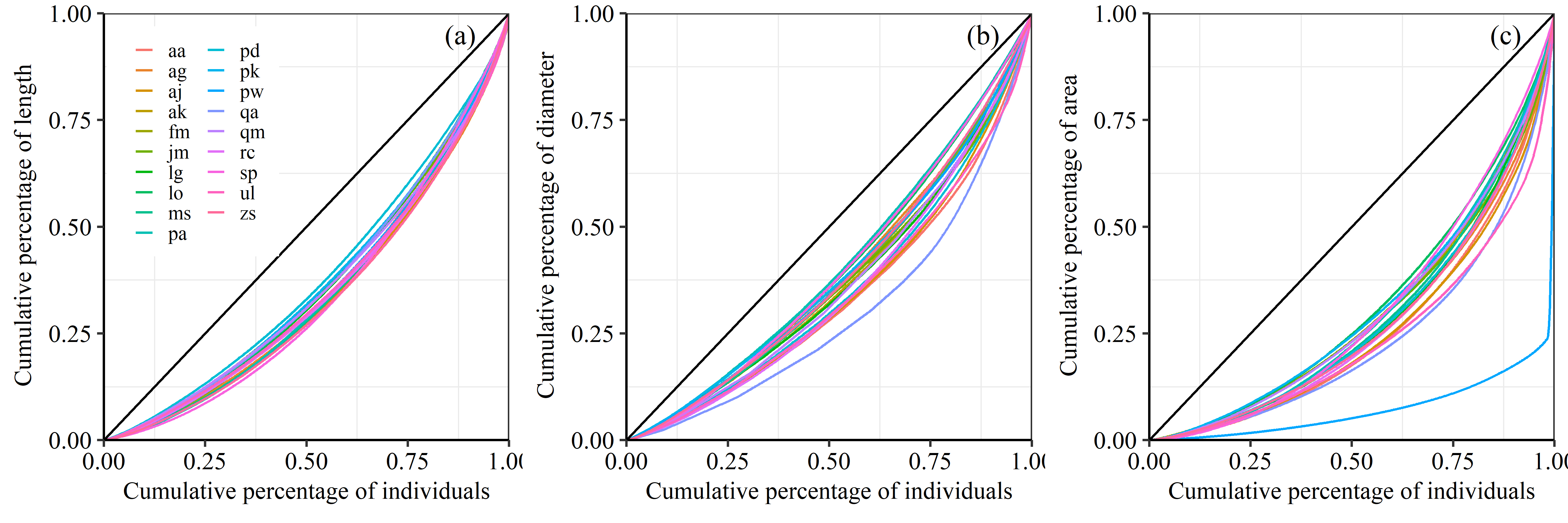


**Supplementary Figure 6.** Lorenz curves of the length (a), diameter (b) and area (c) of first-order fine roots across tree species from the Xianrendong forest. Abbreviations for tree species are given in Table S1.


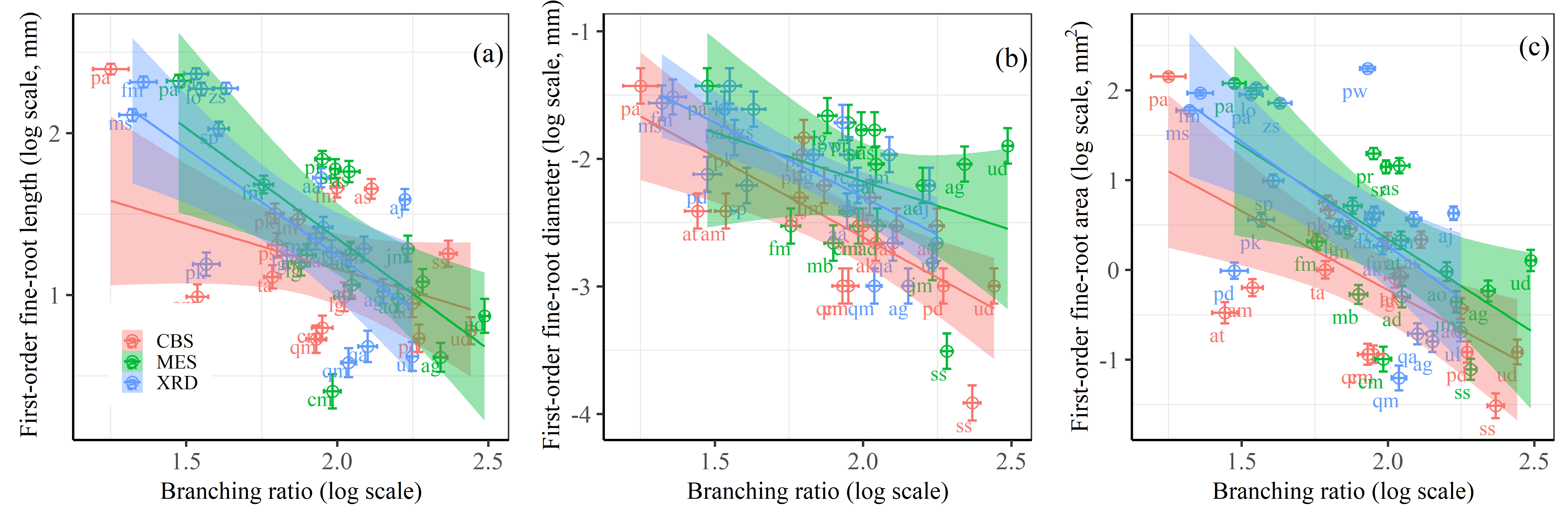


**Supplementary Figure 7.** Relationships between branching ratio and the length (a), diameter (b) and area (c) of first-order fine roots for Changbai Mountain (CBS), Maoershan (MES) and Xianrendong (XRD) forests. Linear mixed-effects kinship model (Table S4) fits are displayed, along with 95% confidence intervals. Abbreviations for tree species are given in Table S1.


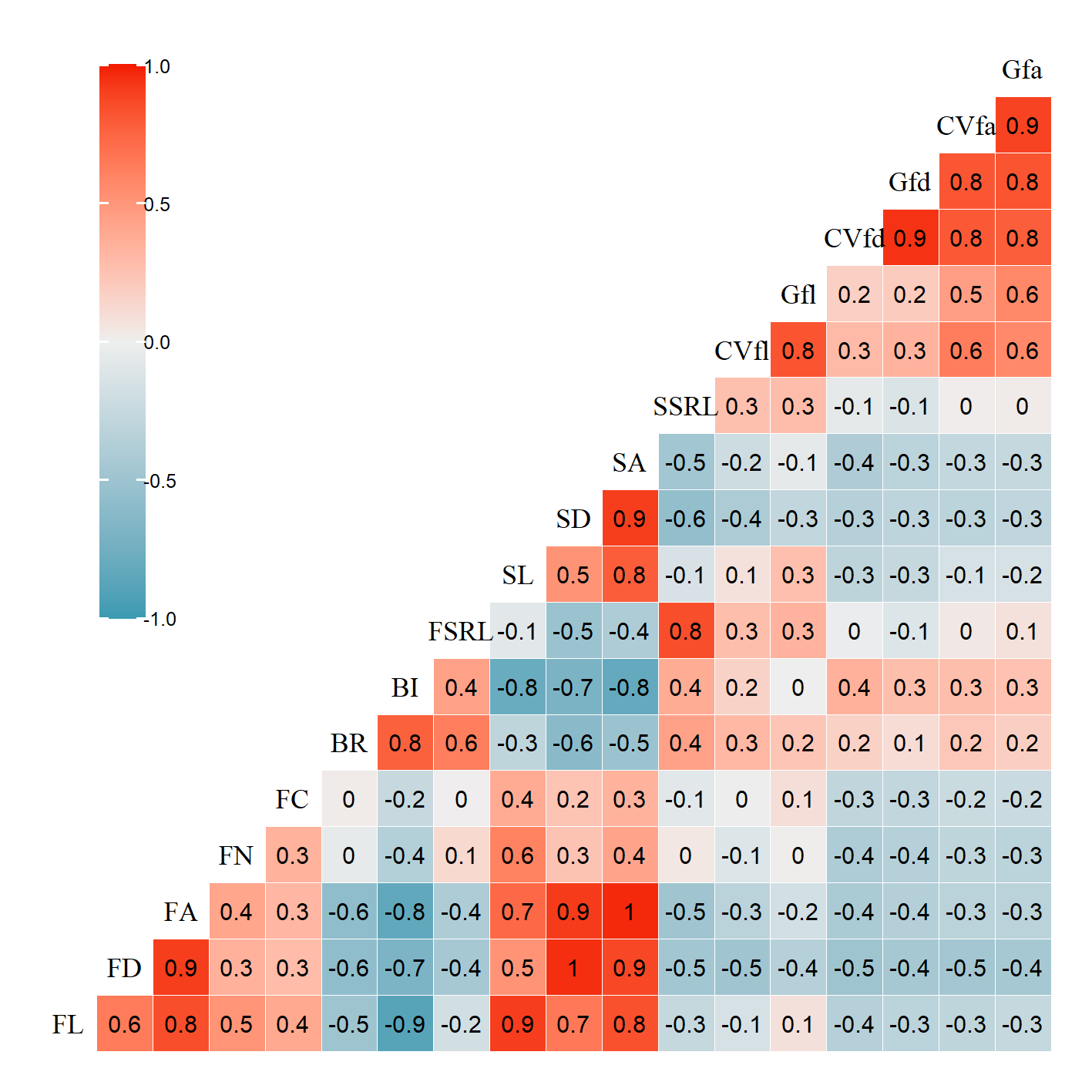


**Supplementary Figure 8.** Relationships between traits for first- and second-order fine roots. Abbreviations for fine-root traits are given in Figure 4.

## Supplementary Tables

**Supplementary Table 1.** Summary of the size and its inequality/variation in first-order fine roots for 51 tree species in Changbai Mountain (CBS), Xianrendong (XRD) and Maoershan (MES) forests.

| Species | Abbr | No | FL | Mi_fl_ | Ma_fl_ | S_fl_ | K_fl_ | CV_fl_ | G_fl_ | FD | Mi_fd_ | Ma_fd_ | S_fd_ | K_fd_ | CV_fd_ | G_fd_ | FA | Mi_fa_ | Ma_fa_ | S_fa_ | K_fa_ | CV_fa_ | G_fa_ |
| --- | --- | --- | --- | --- | --- | --- | --- | --- | --- | --- | --- | --- | --- | --- | --- | --- | --- | --- | --- | --- | --- | --- | --- |
| Changbai Mountain forest (CBS) | | | | | | | | | | | | | | | | | | | | | | | |
| *Albizia kalkora* | ak | 578 | 3.48±0.10 | 0.47 | 21.3 | 2.06 | 6.69 | 0.72 | 0.27 | 0.07±0.01 | 0.01 | 0.26 | 0.95 | 1.43 | 0.57 | 0.51 | 0.93±0.04 | 0.02 | 9.37 | 2.86 | 13.3 | 1.11 | 0.51 |
| *Acer mandshuricum* | am | 331 | 2.69±0.08 | 0.12 | 15.2 | 2.78 | 18.6 | 0.52 | 0.23 | 0.09±0.01 | 0.01 | 0.60 | 4.45 | 47.2 | 0.44 | 0.37 | 0.82±0.04 | 0.06 | 7.65 | 4.80 | 37.4 | 0.88 | 0.37 |
| *Acer mono* | ao | 709 | 2.58±0.06 | 0.51 | 18.6 | 2.80 | 17.6 | 0.62 | 0.20 | 0.08±0.01 | 0.01 | 0.16 | 0.45 | -0.22 | 0.38 | 0.41 | 0.65±0.02 | 0.02 | 6.43 | 3.27 | 21.1 | 0.88 | 0.41 |
| *Acer tegmentosum* | at | 317 | 2.20±0.06 | 0.24 | 7.91 | 1.66 | 4.13 | 0.52 | 0.21 | 0.09±0.01 | 0.01 | 0.21 | 0.79 | 0.96 | 0.33 | 0.35 | 0.62±0.02 | 0.01 | 3.23 | 1.95 | 6.53 | 0.68 | 0.35 |
| *Corylus mandshurica* | cm | 527 | 2.22±0.08 | 0.25 | 32.6 | 8.34 | 118 | 0.87 | 0.31 | 0.05±0.01 | 0.01 | 0.21 | 1.34 | 2.68 | 0.60 | 0.53 | 0.39±0.04 | 0.02 | 21.5 | 18.1 | 376 | 2.56 | 0.53 |
| *Eleutherococcus senticosus* | es | 620 | 5.24±0.13 | 0.85 | 24.4 | 1.75 | 4.71 | 0.63 | 0.18 | 0.08±0.01 | 0.02 | 0.18 | 0.44 | -0.08 | 0.38 | 0.42 | 1.40±0.05 | 0.12 | 13.5 | 3.17 | 18.2 | 0.91 | 0.42 |
| *Fraxinus mandschurica* | fm | 554 | 5.28±0.13 | 0.79 | 27.0 | 1.60 | 5.10 | 0.59 | 0.21 | 0.08±0.01 | 0.03 | 0.19 | 1.04 | 1.40 | 0.38 | 0.42 | 1.38±0.05 | 0.12 | 11.0 | 2.96 | 12.9 | 0.92 | 0.42 |
| *Juglans mandshurica* | jm | 490 | 4.34±0.15 | 0.35 | 52.6 | 7.80 | 101 | 0.75 | 0.19 | 0.11±0.01 | 0.04 | 0.23 | 0.37 | -0.01 | 0.36 | 0.37 | 1.58±0.06 | 0.13 | 17.1 | 5.53 | 51.3 | 0.87 | 0.37 |
| *Larix gmelinii* | lg | 567 | 2.69±0.05 | 0.01 | 8.65 | 1.11 | 2.38 | 0.49 | 0.20 | 0.10±0.01 | 0.01 | 0.26 | 0.62 | 0.80 | 0.40 | 0.36 | 0.91±0.03 | 0.00 | 3.75 | 1.54 | 3.05 | 0.70 | 0.36 |
| *Phellodendron amurense* | pa | 262 | 11.0±0.34 | 1.81 | 39.4 | 0.89 | 1.95 | 0.50 | 0.12 | 0.24±0.01 | 0.10 | 0.37 | -0.02 | -0.22 | 0.21 | 0.33 | 8.63±0.33 | 0.95 | 42.1 | 1.45 | 5.17 | 0.61 | 0.33 |
| *Pinus densiflora* | pd | 726 | 2.08±0.06 | 0.24 | 14.5 | 2.91 | 13.5 | 0.74 | 0.35 | 0.05±0.01 | 0.01 | 0.48 | 4.38 | 33.2 | 0.80 | 0.57 | 0.40±0.02 | 0.01 | 6.83 | 4.73 | 30.1 | 1.60 | 0.57 |
| *Pinus koraiensis* | pk | 451 | 4.50±0.13 | 0.02 | 19.3 | 1.48 | 3.03 | 0.62 | 0.16 | 0.14±0.01 | 0.02 | 0.31 | 0.28 | 0.62 | 0.29 | 0.39 | 2.12±0.08 | 0.00 | 11.0 | 1.87 | 4.83 | 0.77 | 0.39 |
| *Picea asperata* | pa | 448 | 3.70±0.08 | 0.55 | 13.4 | 1.25 | 2.91 | 0.45 | 0.17 | 0.16±0.01 | 0.05 | 0.33 | 0.34 | 0.23 | 0.31 | 0.31 | 1.94±0.05 | 0.21 | 7.14 | 1.27 | 2.44 | 0.57 | 0.31 |
| *Quercus mongolica* | qm | 517 | 2.07±0.06 | 0.08 | 19.2 | 5.27 | 50.3 | 0.69 | 0.27 | 0.05±0.01 | 0.01 | 0.25 | 1.92 | 8.86 | 0.60 | 0.47 | 0.39±0.02 | 0.02 | 7.82 | 8.69 | 99.5 | 1.44 | 0.47 |
| *Sorbaria sorbifolia* | ss | 810 | 3.51±0.07 | 0.59 | 14.8 | 1.37 | 2.44 | 0.60 | 0.20 | 0.02±0.01 | 0.01 | 0.05 | 0.66 | 0.62 | 0.50 | 0.38 | 0.22±0.01 | 0.02 | 1.01 | 1.59 | 3.04 | 0.73 | 0.38 |
| *Tilia amurensis* | ta | 449 | 3.04±0.09 | 0.47 | 14.6 | 1.68 | 4.98 | 0.60 | 0.26 | 0.10±0.01 | 0.02 | 0.47 | 2.41 | 14. 9 | 0.50 | 0.44 | 1.00±0.04 | 0.06 | 7.78 | 2.27 | 9.66 | 0.88 | 0.44 |
| *Ulmus davidiana* | ud | 862 | 2.18±0.06 | 0.17 | 17.3 | 2.60 | 13.2 | 0.75 | 0.27 | 0.05±0.01 | 0.01 | 0.16 | 0.27 | 0.02 | 0.40 | 0.49 | 0.40±0.01 | 0.01 | 3.95 | 2.97 | 14.5 | 1.05 | 0.49 |
| Maoershan forest (MES) | | | | | | | | | | | | | | | | | | | | | | | |
| *Amygdalus davidiana* | ad | 705 | 2.89±0.06 | 0.49 | 9.92 | 1.56 | 3.42 | 0.54 | 0.18 | 0.08±0.01 | 0.02 | 0.19 | 0.68 | 0.95 | 0.38 | 0.37 | 0.74±0.02 | 0.06 | 4.34 | 2.38 | 8.34 | 0.77 | 0.37 |
| *Acer ginnala* | ag | 801 | 1.85±0.05 | 0.23 | 12.0 | 2.15 | 8.21 | 0.71 | 0.14 | 0.13±0.01 | 0.05 | 0.28 | 0.51 | 0.32 | 0.23 | 0.41 | 0.79±0.02 | 0.05 | 6.54 | 2.77 | 12.7 | 0.87 | 0.41 |
| *Acer mono* | ao | 680 | 2.66±0.06 | 0.40 | 12.1 | 1.89 | 5.52 | 0.56 | 0.16 | 0.11±0.01 | 0.01 | 0.23 | 0.21 | 0.27 | 0.27 | 0.35 | 0.98±0.03 | 0.03 | 5.29 | 1.95 | 6.16 | 0.69 | 0.35 |
| *Acer tegmentosum* | at | 577 | 3.50±0.08 | 0.60 | 16.0 | 1.84 | 5.19 | 0.57 | 0.15 | 0.13±0.01 | 0.01 | 0.26 | 0.30 | 0.39 | 0.23 | 0.32 | 1.39±0.04 | 0.07 | 7.05 | 2.15 | 6.76 | 0.66 | 0.32 |
| *Corylus mandshurica* | cm | 545 | 1.50±0.03 | 0.24 | 8.14 | 1.85 | 10.0 | 0.51 | 0.16 | 0.08±0.01 | 0.02 | 0.52 | 7.30 | 116 | 0.38 | 0.33 | 0.37±0.01 | 0.02 | 2.69 | 2.73 | 16. 7 | 0.68 | 0.33 |
| *Eleutherococcus senticosus* | es | 563 | 5.83±0.11 | 1.07 | 17.5 | 0.85 | 1.25 | 0.44 | 0.09 | 0.17±0.01 | 0.09 | 0.29 | 0.58 | 1.24 | 0.18 | 0.26 | 3.19±0.06 | 0.37 | 11.0 | 0.90 | 1.52 | 0.48 | 0.26 |
| *Fraxinus mandschurica* | fm | 424 | 5.38±0.16 | 0.75 | 20.3 | 1.39 | 2.42 | 0.60 | 0.18 | 0.08±0.01 | 0.02 | 0.16 | 0.48 | 0.42 | 0.25 | 0.39 | 1.37±0.05 | 0.06 | 6.64 | 1.71 | 3.41 | 0.77 | 0.39 |
| *Juglans mandshurica* | jm | 708 | 3.62±0.07 | 0.64 | 12.6 | 1.26 | 2.09 | 0.54 | 0.20 | 0.06±0.01 | 0.02 | 0.26 | 1.45 | 8.61 | 0.33 | 0.37 | 0.70±0.02 | 0.05 | 3.98 | 2.11 | 7.00 | 0.76 | 0.37 |
| *Larix gmelinii* | lg | 502 | 3.32±0.07 | 0.88 | 9.71 | 1.21 | 1.69 | 0.47 | 0.12 | 0.19±0.01 | 0.10 | 0.34 | 0.25 | -0.01 | 0.21 | 0.28 | 2.04±0.05 | 0.32 | 6.31 | 1.18 | 1.42 | 0.52 | 0.28 |
| *Malus baccata* | mb | 499 | 3.48±0.10 | 0.40 | 13.4 | 1.45 | 2.13 | 0.66 | 0.17 | 0.07±0.01 | 0.02 | 0.15 | 0.73 | 0.94 | 0.29 | 0.41 | 0.76±0.03 | 0.06 | 4.36 | 2.12 | 5.89 | 0.84 | 0.41 |
| *Phellodendron amurense* | pa | 321 | 10.2±0.28 | 0.80 | 25.8 | 0.72 | 0.03 | 0.49 | 0.12 | 0.24±0.01 | 0.08 | 0.37 | -0.22 | -0.06 | 0.21 | 0.31 | 7.98±0.25 | 0.20 | 25.1 | 0.87 | 0.60 | 0.57 | 0.31 |
| *Padus racemose* | pr | 534 | 6.30±0.19 | 0.49 | 26. 9 | 1.52 | 2.65 | 0.69 | 0.10 | 0.18±0.01 | 0.05 | 0.32 | 0.50 | 2.12 | 0.17 | 0.40 | 3.65±0.13 | 0.20 | 19.1 | 1.99 | 5.42 | 0.80 | 0.40 |
| *Syringa reticulate* | sr | 558 | 5.93±0.14 | 0.64 | 20.9 | 0.91 | 1.00 | 0.57 | 0.13 | 0.17±0.01 | 0.04 | 0.27 | 0.13 | 0.35 | 0.24 | 0.36 | 3.15±0.09 | 0.11 | 12.4 | 1.15 | 1.54 | 0.66 | 0.36 |
| *Sorbaria sorbifolia* | ss | 735 | 2.95±0.07 | 0.09 | 15.1 | 1.53 | 4.33 | 0.62 | 0.28 | 0.03±0.01 | 0.01 | 0.71 | 10.8 | 139 | 1.33 | 0.46 | 0.33±0.02 | 0.01 | 7.20 | 8.05 | 98.4 | 1.33 | 0.46 |
| *Ulmus davidiana* | ud | 1051 | 2.39±0.03 | 0.48 | 8.43 | 1.21 | 2.52 | 0.45 | 0.10 | 0.15±0.01 | 0.05 | 0.22 | -0.07 | 0.65 | 0.20 | 0.27 | 1.11±0.02 | 0.13 | 3.97 | 1.24 | 2.32 | 0.50 | 0.27 |

**Supplementary Table 1.** continued

| Species | Abbr | No | FL | Mi_fl_ | Ma_fl_ | S_fl_ | K_fl_ | CV_fl_ | G_fl_ | FD | Mi_fd_ | Ma_fd_ | S_fd_ | K_fd_ | CV_fd_ | G_fd_ | FA | Mi_fa_ | Ma_fa_ | S_fa_ | K_fa_ | CV_fa_ | G_fa_ |
| --- | --- | --- | --- | --- | --- | --- | --- | --- | --- | --- | --- | --- | --- | --- | --- | --- | --- | --- | --- | --- | --- | --- | --- |
| Xianrendong forest (XRD) | | | | | | | | | | | | | | | | | | | | | | | |
| *Actinidia argute* | aa | 533 | 5.61±0.14 | 0.56 | 24.2 | 1.22 | 2.64 | 0.57 | 0.33 | 0.09±0.01 | 0.02 | 0.48 | 2.17 | 5.61 | 0.67 | 0.49 | 1.76±0.08 | 0.05 | 13.4 | 2.47 | 7.33 | 1.06 | 0.49 |
| *Acer ginnala* | ag | 645 | 2.78±0.08 | 0.53 | 21.9 | 3.41 | 21.4 | 0.70 | 0.23 | 0.05±0.01 | 0.01 | 0.19 | 2.21 | 8.20 | 0.40 | 0.45 | 0.45±0.02 | 0.01 | 9.61 | 8.22 | 108 | 1.29 | 0.45 |
| *Alnus japonica* | aj | 693 | 4.91±0.12 | 0.03 | 20.2 | 1.76 | 4.18 | 0.64 | 0.32 | 0.11±0.01 | 0.02 | 0.44 | 1.56 | 2.16 | 0.64 | 0.49 | 1.88±0.08 | 0.01 | 14.6 | 3.00 | 10.6 | 1.14 | 0.49 |
| *Albizia kalkora* | ak | 544 | 3.54±0.08 | 0.68 | 12.2 | 1.27 | 2.22 | 0.50 | 0.26 | 0.11±0.01 | 0.02 | 0.38 | 1.55 | 2.30 | 0.55 | 0.40 | 1.30±0.05 | 0.09 | 8.71 | 2.38 | 8.21 | 0.84 | 0.40 |
| *Fraxinus mandschurica* | fm | 288 | 10.1±0.33 | 1.03 | 49.6 | 2.06 | 9.62 | 0.55 | 0.24 | 0.22±0.01 | 0.08 | 1.03 | 3.19 | 18.7 | 0.50 | 0.40 | 7.17±0.36 | 0.62 | 59.2 | 3.16 | 18.8 | 0.85 | 0.40 |
| *Juglans mandshurica* | jm | 606 | 3.62±0.08 | 0.81 | 12.7 | 1.61 | 3.59 | 0.52 | 0.25 | 0.14±0.01 | 0.04 | 0.44 | 1.72 | 2.50 | 0.50 | 0.41 | 1.76±0.07 | 0.10 | 12.8 | 3.14 | 12.8 | 0.95 | 0.41 |
| *Larix gmelinii* | lg | 469 | 3.47±0.08 | 0.55 | 10.6 | 1.05 | 1.28 | 0.51 | 0.26 | 0.14±0.01 | 0.03 | 0.36 | 1.12 | 0.22 | 0.50 | 0.44 | 1.62±0.07 | 0.10 | 9.40 | 1.86 | 4.12 | 0.88 | 0.44 |
| *Lindera obtusiloba* | lo | 353 | 9.71±0.27 | 1.30 | 44.1 | 1.53 | 5.88 | 0.52 | 0.20 | 0.24±0.01 | 0.07 | 0.52 | 0.67 | 0.00 | 0.33 | 0.37 | 7.61±0.31 | 0.71 | 65.1 | 3.47 | 25.4 | 0.78 | 0.37 |
| *Magnolia sieboldii;* | ms | 281 | 8.27±0.29 | 1.19 | 26.9 | 1.07 | 0.82 | 0.58 | 0.24 | 0.21±0.01 | 0.03 | 0.50 | 0.89 | 0.45 | 0.43 | 0.42 | 5.91±0.30 | 0.11 | 31.5 | 1.82 | 4.03 | 0.85 | 0.42 |
| *Phellodendron amurense* | pa | 346 | 10.7±0.33 | 1.10 | 32.1 | 1.07 | 1.05 | 0.58 | 0.18 | 0.20±0.01 | 0.02 | 0.39 | 0.40 | -0.31 | 0.30 | 0.40 | 7.04±0.30 | 0.14 | 30.9 | 1.73 | 3.69 | 0.79 | 0.40 |
| *Pinus densiflora* | pd | 328 | 2.35±0.06 | 0.64 | 12.4 | 2.83 | 18.1 | 0.49 | 0.24 | 0.12±0.01 | 0.02 | 0.50 | 1.66 | 3.17 | 0.58 | 0.85 | 0.99±0.05 | 0.08 | 9.71 | 3.38 | 21.4 | 0.96 | 0.85 |
| *Pinus koraiensis* | pk | 358 | 3.29±0.09 | 0.48 | 18.9 | 2.64 | 17.2 | 0.53 | 0.30 | 0.16±0.01 | 0.06 | 0.47 | 1.58 | 2.22 | 0.44 | 0.44 | 1.75±0.08 | 0.12 | 14.3 | 3.13 | 16.7 | 0.84 | 0.44 |
| *Picea wilsonii* | pw | 511 | 3.86±0.10 | 0.71 | 25.8 | 2.70 | 16.4 | 0.61 | 0.23 | 0.18±0.01 | 0.04 | 0.55 | 1.33 | 1.50 | 0.44 | 0.39 | 9.41±2.79 | 0.10 | 804 | 9.85 | 102 | 6.70 | 0.39 |
| *Quercus acutissima* | qa | 632 | 1.98±0.04 | 0.41 | 7.29 | 1.32 | 2.70 | 0.51 | 0.40 | 0.07±0.01 | 0.01 | 0.35 | 1.73 | 2.57 | 0.86 | 0.52 | 0.49±0.02 | 0.03 | 4.20 | 3.03 | 11.0 | 1.24 | 0.52 |
| *Quercus mongolica* | qm | 566 | 1.79±0.05 | 0.35 | 12.7 | 3.74 | 24.1 | 0.63 | 0.27 | 0.05±0.01 | 0.01 | 0.17 | 1.53 | 2.75 | 0.60 | 0.40 | 0.30±0.01 | 0.02 | 3.58 | 4.45 | 40.3 | 0.90 | 0.40 |
| *Rhus chinensis* | rc | 529 | 4.13±0.11 | 0.72 | 13.8 | 1.50 | 2.33 | 0.60 | 0.30 | 0.14±0.01 | 0.02 | 0.45 | 1.25 | 1.80 | 0.57 | 0.45 | 1.88±0.08 | 0.10 | 11.1 | 2.16 | 5.69 | 0.94 | 0.45 |
| *Symplocos paniculata* | sp | 374 | 7.59±0.23 | 1.12 | 22.7 | 0.72 | 0.03 | 0.58 | 0.20 | 0.11±0.01 | 0.02 | 0.23 | 0.31 | -0.08 | 0.36 | 0.39 | 2.70±0.10 | 0.10 | 11.4 | 1.02 | 1.13 | 0.72 | 0.39 |
| *Ulmus laciniata* | ul | 713 | 1.86±0.05 | 0.33 | 9.61 | 2.63 | 9.84 | 0.65 | 0.32 | 0.07±0.01 | 0.01 | 0.41 | 3.04 | 11.2 | 0.86 | 0.52 | 0.50±0.03 | 0.02 | 6.07 | 4.32 | 21.8 | 1.48 | 0.52 |
| *Zanthoxylum simulans* | zs | 383 | 9.75±0.32 | 0.24 | 36.0 | 1.44 | 2.26 | 0.64 | 0.23 | 0.20±0.01 | 0.03 | 0.71 | 1.37 | 3.73 | 0.45 | 0.45 | 6.41±0.31 | 0.45 | 38.1 | 2.20 | 5.89 | 0.94 | 0.45 |

Abbr, abbreviation of species; No, number of individual first-order fine roots; FL, first-order fine-root length; Mi_fl_, minimum value of FL; Ma_fl_, maximum value of FL; S_fl_, skewness of FL; K_fl_, kurtosis of FL; CV_fl_, coefficient of variation of FL; G_fl_, Gini coefficient of FL; FD, first-order fine-root diameter; Mi_fd_, minimum value of FD; Ma_fd_, maximum value of FD; S_fd_, skewness of FD; K_fd_, kurtosis of FD; CV_fd_, coefficient of variation of FD; G_fd_, Gini coefficient of FD; FA, first-order fine-root area; Mi_fa_, minimum value of FA; Ma_fa_, maximum value of FA; S_fa_, skewness of FA; K_fa_, kurtosis of FA; CV_fa_, coefficient of variation of FA; G_fa_, Gini coefficient of FA.

**Supplementary Table 2.** Summary of the size and its inequality/variation in second-order fine roots for 51 tree species in Changbai Mountain (CBS), Xianrendong (XRD) and Maoershan (MES) forests.

| Species | Abbr | No | SL | Mi_sl_ | Ma_sl_ | S_sl_ | K_sl_ | CV_sl_ | SD | Mi_sd_ | Ma_sd_ | S_sd_ | K_sd_ | CV_sd_ | SA | Mi_sa_ | Ma_sa_ | S_sa_ | K_sa_ | CV_sa_ |
| --- | --- | --- | --- | --- | --- | --- | --- | --- | --- | --- | --- | --- | --- | --- | --- | --- | --- | --- | --- | --- |
| Changbai Mountain forest (CBS) | | | | | | | | | | | | | | | | | | | | |
| *Albizia kalkora* | ak | 75 | 19.2±1.02 | 4.90 | 48.2 | 0.93 | 0.47 | 0.46 | 0.16±0.01 | 0.06 | 0.30 | 0.26 | -0.82 | 0.38 | 10.7±0.99 | 1.54 | 43.9 | 1.64 | 2.78 | 0.80 |
| *Acer mandshuricum* | am | 75 | 14.5±0.80 | 4.37 | 48.3 | 2.19 | 6.89 | 0.47 | 0.16±0.01 | 0.01 | 0.27 | -0.81 | 1.73 | 0.31 | 7.42±0.56 | 0.84 | 30.4 | 2.30 | 7.63 | 0.65 |
| *Acer mono* | ao | 75 | 16.3±0.93 | 7.15 | 56.7 | 2.30 | 7.31 | 0.49 | 0.13±0.01 | 0.06 | 0.23 | 0.46 | 0.26 | 0.23 | 6.98±0.52 | 2.20 | 32.0 | 2.55 | 10.8 | 0.64 |
| *Acer tegmentosum* | at | 75 | 11.2±1.20 | 2.31 | 91.3 | 6.10 | 44.0 | 0.93 | 0.14±0.01 | 0.07 | 0.26 | 0.27 | -0.12 | 0.29 | 5.01±0.42 | 0.93 | 25.8 | 2.83 | 12.3 | 0.73 |
| *Corylus mandshurica* | cm | 75 | 14.2±0.67 | 5.95 | 34.4 | 1.48 | 2.36 | 0.41 | 0.11±0.01 | 0.01 | 0.21 | 0.37 | -0.36 | 0.36 | 5.28±0.45 | 0.38 | 17.3 | 1.65 | 2.32 | 0.74 |
| *Eleutherococcus senticosus* | es | 75 | 22.5±1.30 | 10.1 | 73.3 | 2.29 | 7.11 | 0.50 | 0.15±0.01 | 0.07 | 0.25 | 0.56 | -0.09 | 0.27 | 10.8±0.96 | 2.21 | 54.7 | 3.24 | 13.0 | 0.77 |
| *Fraxinus mandschurica* | fm | 73 | 21.1±0.89 | 9.36 | 44.3 | 0.86 | 0.53 | 0.36 | 0.16±0.01 | 0.06 | 0.35 | 1.01 | 0.33 | 0.38 | 11.3±0.96 | 2.83 | 45.0 | 2.11 | 4.89 | 0.72 |
| *Juglans mandshurica* | jm | 75 | 22.2±1.00 | 9.18 | 47.3 | 0.82 | 0.21 | 0.39 | 0.17±0.01 | 0.09 | 0.47 | 2.18 | 9.67 | 0.35 | 12.0±0.74 | 3.17 | 33.0 | 1.11 | 0.93 | 0.54 |
| *Larix gmelinii* | lg | 75 | 13.5±0.58 | 6.94 | 31.6 | 1.64 | 2.90 | 0.37 | 0.19±0.01 | 0.12 | 0.37 | 1.15 | 2.30 | 0.21 | 8.24±0.41 | 3.53 | 19.8 | 1.10 | 0.66 | 0.43 |
| *Phellodendron amurense* | pa | 75 | 31.0±1.24 | 12.9 | 57.7 | 0.55 | -0.33 | 0.35 | 0.33±0.01 | 0.21 | 0.49 | 0.28 | 0.00 | 0.15 | 32.5±1.53 | 13.7 | 88.8 | 1.23 | 2.83 | 0.41 |
| *Pinus densiflora* | pd | 75 | 14.4±0.84 | 3.47 | 37.8 | 1.20 | 1.52 | 0.51 | 0.13±0.01 | 0.04 | 0.31 | 1.00 | 0.29 | 0.54 | 6.68±0.72 | 0.44 | 33.2 | 1.94 | 4.24 | 0.94 |
| *Pinus koraiensis* | pk | 75 | 19.9±0.89 | 7.89 | 44.1 | 1.04 | 0.67 | 0.39 | 0.22±0.01 | 0.02 | 0.36 | -0.14 | 0.51 | 0.27 | 14.5±0.92 | 0.94 | 45.7 | 1.33 | 2.48 | 0.55 |
| *Picea asperata* | pa | 75 | 18.2±0.97 | 6.01 | 51.6 | 1.39 | 2.61 | 0.46 | 0.28±0.01 | 0.13 | 0.44 | 0.13 | -0.21 | 0.25 | 16.3±1.15 | 3.04 | 61.6 | 1.79 | 4.75 | 0.61 |
| *Quercus mongolica* | qm | 75 | 12.2±0.48 | 4.04 | 25.7 | 0.76 | 0.84 | 0.34 | 0.14±0.01 | 0.05 | 0.30 | 0.98 | 1.98 | 0.36 | 5.56±0.32 | 1.09 | 12.3 | 0.86 | 0.16 | 0.50 |
| *Sorbaria sorbifolia* | ss | 76 | 19.9±0.71 | 10.3 | 38.4 | 0.58 | -0.11 | 0.31 | 0.07±0.01 | 0.03 | 0.11 | 0.47 | -0.25 | 0.29 | 4.18±0.24 | 1.34 | 13.3 | 1.52 | 3.64 | 0.50 |
| *Tilia amurensis* | ta | 75 | 16.4±0.80 | 4.26 | 35. 9 | 0.50 | -0.13 | 0.43 | 0.18±0.01 | 0.05 | 0.37 | 0.27 | -0.34 | 0.39 | 10.3±0.81 | 1.05 | 32.3 | 1.09 | 0.92 | 0.68 |
| *Ulmus davidiana* | ud | 75 | 18.1±1.10 | 2.15 | 42.7 | 0.26 | -0.66 | 0.53 | 0.10±0.01 | 0.01 | 0.18 | -0.32 | -0.67 | 0.40 | 6.25±0.51 | 0.12 | 15.9 | 0.47 | -0.66 | 0.71 |
| Maoershan forest (MES) | | | | | | | | | | | | | | | | | | | | |
| *Amygdalus davidiana* | ad | 90 | 13.6±0.53 | 4.99 | 34.8 | 1.73 | 4.09 | 0.37 | 0.13±0.01 | 0.07 | 0.20 | 0.50 | -0.51 | 0.23 | 5.53±0.31 | 1.57 | 17.5 | 2.08 | 5.62 | 0.52 |
| *Acer ginnala* | ag | 75 | 16.6±0.68 | 7.58 | 34.9 | 0.88 | 0.37 | 0.35 | 0.20±0.01 | 0.12 | 0.36 | 0.66 | 1.51 | 0.20 | 10.7±0.51 | 2.86 | 24.6 | 0.89 | 0.50 | 0.42 |
| *Acer mono* | ao | 75 | 15.7±0.66 | 8.18 | 36.1 | 1.04 | 1.12 | 0.36 | 0.16±0.01 | 0.08 | 0.28 | 0.16 | -0.25 | 0.25 | 8.28±0.48 | 2.14 | 21.9 | 1.15 | 0.96 | 0.50 |
| *Acer tegmentosum* | at | 75 | 17.6±0.83 | 5.55 | 39.1 | 1.32 | 1.76 | 0.41 | 0.19±0.01 | 0.09 | 0.29 | 0.08 | -0.50 | 0.21 | 10.5±0.62 | 3.11 | 30.6 | 1.46 | 2.50 | 0.51 |
| *Corylus mandshurica* | cm | 75 | 9.23±0.45 | 2.97 | 24.8 | 1.12 | 1.89 | 0.42 | 0.11±0.01 | 0.04 | 0.17 | -0.09 | 2.11 | 0.18 | 3.27±0.22 | 0.53 | 13.2 | 2.34 | 9.08 | 0.57 |
| *Eleutherococcus senticosus* | es | 73 | 17.8±0.76 | 5.19 | 40.2 | 0.85 | 0.96 | 0.37 | 0.26±0.01 | 0.17 | 0.44 | 1.48 | 2.78 | 0.19 | 14.4±0.69 | 3.42 | 29.5 | 0.71 | 0.21 | 0.41 |
| *Fraxinus mandschurica* | fm | 74 | 20.0±0.75 | 9.78 | 42.4 | 0.97 | 0.72 | 0.32 | 0.13±0.01 | 0.07 | 0.27 | 1.10 | 1.80 | 0.31 | 8.64±0.59 | 3.07 | 36.0 | 2.69 | 10.7 | 0.59 |
| *Juglans mandshurica* | jm | 76 | 20.5±0.98 | 8.70 | 39.7 | 0.48 | -0.93 | 0.42 | 0.10±0.01 | 0.05 | 0.17 | 0.39 | -0.55 | 0.30 | 6.51±0.44 | 1.64 | 16.0 | 0.79 | -0.62 | 0.60 |
| *Larix gmelinii* | lg | 76 | 16.1±0.67 | 6.04 | 35.2 | 0.74 | 0.97 | 0.36 | 0.28±0.01 | 0.18 | 0.36 | -0.21 | 0.70 | 0.11 | 14.0±0.59 | 5.23 | 28.8 | 0.64 | 0.14 | 0.37 |
| *Malus baccata* | mb | 75 | 18.1±0.70 | 6.79 | 38.4 | 0.70 | 0.76 | 0.33 | 0.11±0.01 | 0.06 | 0.21 | 0.94 | 0.94 | 0.27 | 6.56±0.37 | 2.12 | 19. 5 | 1.59 | 3.67 | 0.49 |
| *Phellodendron amurense* | pa | 73 | 32.0±1.46 | 10.9 | 70.4 | 0.81 | 0.30 | 0.39 | 0.35±0.01 | 0.20 | 0.47 | -0.22 | -0.43 | 0.17 | 36.0±2.12 | 6.86 | 99.5 | 1.21 | 1.68 | 0.50 |
| *Padus racemose* | pr | 74 | 22.8±1.00 | 7.30 | 56.4 | 1.06 | 2.09 | 0.38 | 0.24±0.01 | 0.16 | 0.43 | 1.64 | 4.32 | 0.21 | 17.7±1.02 | 3.67 | 54.0 | 1.70 | 4.25 | 0.49 |
| *Syringa reticulate* | sr | 76 | 22.3±0.84 | 9.78 | 45.9 | 0.91 | 0.80 | 0.33 | 0.26±0.01 | 0.11 | 0.42 | 0.11 | 0.01 | 0.23 | 19.0±1.17 | 5.84 | 60.5 | 1.48 | 2.80 | 0.53 |
| *Sorbaria sorbifolia* | ss | 74 | 16.0±1.91 | 0.85 | 150 | 7.36 | 57.4 | 1.03 | 0.09±0.01 | 0.03 | 0.18 | 0.32 | 1.71 | 0.33 | 4.50±0.63 | 0.08 | 47.1 | 6.74 | 50.3 | 1.20 |
| *Ulmus davidiana* | ud | 88 | 17.8±0.71 | 6.81 | 35.1 | 0.65 | -0.58 | 0.38 | 0.19±0.01 | 0.12 | 0.27 | 0.26 | 0.97 | 0.16 | 10.9±0.46 | 3.41 | 20.9 | 0.43 | -0.68 | 0.40 |

**Supplementary Table 2.** continued

| Species | Abbr | No | SL | Mi_sl_ | Ma_sl_ | S_sl_ | K_sl_ | CV_sl_ | SD | Mi_sd_ | Ma_sd_ | S_sd_ | K_sd_ | CV_sd_ | SA | Mi_sa_ | Ma_sa_ | S_sa_ | K_sa_ | CV_sa_ |
| --- | --- | --- | --- | --- | --- | --- | --- | --- | --- | --- | --- | --- | --- | --- | --- | --- | --- | --- | --- | --- |
| Xianrendong forest (XRD) | | | | | | | | | | | | | | | | | | | | |
| *Actinidia argute* | aa | 76 | 23.4±1.03 | 7.28 | 55.3 | 1.39 | 2.78 | 0.38 | 0.17±0.01 | 0.05 | 0.53 | 1.75 | 5.82 | 0.41 | 13.4±1.19 | 2.06 | 63.6 | 2.64 | 8.19 | 0.78 |
| *Acer ginnala* | ag | 75 | 18.5±0.85 | 7.42 | 43.0 | 1.37 | 1.78 | 0.40 | 0.11±0.01 | 0.05 | 0.27 | 1.46 | 4.79 | 0.36 | 6.32±0.41 | 1.16 | 16.4 | 1.10 | 0.55 | 0.56 |
| *Alnus japonica* | aj | 75 | 22.6±0.90 | 8.53 | 41.2 | 0.37 | -0.42 | 0.34 | 0.23±0.01 | 0.06 | 0.44 | 0.31 | 0.10 | 0.30 | 16.7±0.99 | 2.95 | 41.8 | 0.73 | 0.01 | 0.51 |
| *Albizia kalkora* | ak | 75 | 19.0±0.96 | 4.44 | 48.6 | 1.29 | 1.69 | 0.44 | 0.17±0.01 | 0.06 | 0.31 | 0.65 | 0.67 | 0.29 | 10.3±0.76 | 2.37 | 47.3 | 2.61 | 11.1 | 0.64 |
| *Fraxinus mandschurica* | fm | 74 | 31.3±1.35 | 9.51 | 71. 7 | 1.00 | 1.59 | 0.37 | 0.31±0.01 | 0.16 | 0.54 | 0.58 | 0.01 | 0.26 | 30.4±1.70 | 7.53 | 69.8 | 1.04 | 0.39 | 0.48 |
| *Juglans mandshurica* | jm | 75 | 19.6±0.99 | 2.27 | 51.2 | 0.87 | 1.23 | 0.44 | 0.20±0.01 | 0.11 | 0.45 | 1.04 | 2.34 | 0.30 | 13.0±0.91 | 0.78 | 44. 8 | 1.47 | 2.98 | 0.60 |
| *Larix gmelinii* | lg | 75 | 17.3±0.66 | 5.88 | 30.4 | 0.41 | -0.58 | 0.33 | 0.23±0.01 | 0.11 | 0.40 | 0.77 | 0.89 | 0.22 | 12.4±0.65 | 3.32 | 31.6 | 1.24 | 1.65 | 0.46 |
| *Lindera obtusiloba* | lo | 75 | 32.2±1.47 | 14.6 | 77.2 | 1.29 | 1.91 | 0.40 | 0.31±0.01 | 0.01 | 0.55 | 0.03 | 3.32 | 0.26 | 31.7±1.95 | 1.21 | 89.7 | 1.18 | 1.04 | 0.53 |
| *Magnolia sieboldii;* | ms | 75 | 27.2±1.06 | 11.8 | 66.7 | 1.45 | 3.84 | 0.34 | 0.31±0.01 | 0.16 | 0.53 | 0.62 | 0.25 | 0.23 | 27.4±1.61 | 8.49 | 103 | 2.49 | 10.2 | 0.51 |
| *Phellodendron amurense* | pa | 75 | 30.2±1.33 | 11.1 | 64.8 | 0.57 | -0.12 | 0.38 | 0.34±0.01 | 0.21 | 0.85 | 1.81 | 5.27 | 0.32 | 33.0±2.31 | 9.43 | 120 | 1.98 | 5.17 | 0.61 |
| *Pinus densiflora* | pd | 75 | 8.91±0.29 | 3.73 | 17.6 | 0.70 | 0.68 | 0.28 | 0.23±0.01 | 0.09 | 0.38 | 0.43 | -0.52 | 0.30 | 6.59±0.35 | 1.05 | 14.8 | 0.77 | 0.12 | 0.46 |
| *Pinus koraiensis* | pk | 75 | 13.2±0.55 | 5.39 | 31.1 | 0.88 | 1.06 | 0.36 | 0.27±0.01 | 0.18 | 0.45 | 0.85 | 0.37 | 0.22 | 11.4±0.63 | 4.13 | 29.3 | 1.33 | 1.71 | 0.48 |
| *Picea wilsonii* | pw | 73 | 20.5±1.42 | 7.24 | 80.5 | 2.38 | 7.74 | 0.59 | 0.30±0.01 | 0.17 | 0.55 | 0.88 | 0.71 | 0.23 | 20.3±1.75 | 4.62 | 98.6 | 2.49 | 8.90 | 0.74 |
| *Quercus acutissima* | qa | 77 | 10.9±0.47 | 3.76 | 24.8 | 1.02 | 0.86 | 0.38 | 0.13±0.01 | 0.03 | 0.46 | 1.58 | 3.34 | 0.62 | 4.71±0.43 | 0.90 | 21.9 | 2.16 | 5.95 | 0.81 |
| *Quercus mongolica* | qm | 75 | 10.3±0.50 | 4.43 | 27.0 | 1.56 | 2.78 | 0.42 | 0.11±0.01 | 0.05 | 0.27 | 1.39 | 1.42 | 0.45 | 3.54±0.22 | 1.02 | 8.55 | 0.86 | -0.22 | 0.55 |
| *Rhus chinensis* | rc | 75 | 18.2±0.81 | 7.26 | 41.3 | 0.84 | 0.75 | 0.39 | 0.21±0.01 | 0.08 | 0.46 | 1.03 | 0.91 | 0.38 | 12.3±0.80 | 2.28 | 36.3 | 1.04 | 0.95 | 0.57 |
| *Symplocos paniculata* | sp | 75 | 28.5±1.12 | 11.4 | 49.0 | 0.02 | -0.82 | 0.34 | 0.20±0.01 | 0.09 | 0.48 | 1.49 | 5.01 | 0.30 | 18.0±1.01 | 3.60 | 42.4 | 0.64 | 0.16 | 0.49 |
| *Ulmus laciniata* | ul | 75 | 11.3±0.83 | 3.67 | 47.8 | 2.51 | 8.15 | 0.64 | 0.13±0.01 | 0.07 | 0.26 | 0.83 | 0.26 | 0.31 | 5.15±0.59 | 0.99 | 27.3 | 2.85 | 8.47 | 0.99 |
| *Zanthoxylum simulans* | zs | 75 | 36.4±1.60 | 10.9 | 86. 7 | 0.84 | 0.86 | 0.38 | 0.30±0.01 | 0.19 | 0.48 | 0.75 | 0.06 | 0.23 | 35.0±2.14 | 9.93 | 92.5 | 1.08 | 0.77 | 0.53 |

Abbr, abbreviation of species; No, number of individual second-order fine-roots; SL, second-order fine-root length; Mi_sl_, minimum value of SL; Ma_sl_, maximum value of SL; S_sl_, skewness of SL; K_sl_, kurtosis of SL; CV_sl_, coefficient of variation of SL; SD, second-order fine-root diameter; Mi_sd_, minimum value of SD; Ma_sd_, maximum value of SD; S_sd_, skewness of SD; K_sd_, kurtosis of SD; CV_sd_, coefficient of variation of SD; SA, second-order fine-root area; Mi_sa_, minimum value of SA; Ma_sa_, maximum value of SA; S_sa_, skewness of SA; K_sa_, kurtosis of SA; CV_sa_, coefficient of variation of FA.

**Supplementary Table 3.** Summary of branching ratio (BR) and intensity (BI), specific root length of first- (FSRL, n = 5) and second-order fine roots (SSRL, n = 5), and nitrogen (FN, n = 5) and carbon concentrations of first-order fine roots (FC, n = 5) for 51 tree species in Changbai Mountain (CBS), Xianrendong (XRD) and Maoershan (MES) forests.

| Species | Abbr | No | BR | Mi_br_ | Ma_br_ | S_br_ | K_br_ | CV_br_ | BI | Mi_bi_ | Ma_bi_ | S_bi_ | K_bi_ | CV_bi_ | FSRL | SSRL | FN | FC |
| --- | --- | --- | --- | --- | --- | --- | --- | --- | --- | --- | --- | --- | --- | --- | --- | --- | --- | --- |
| Changbai Mountain forest (CBS) | | | | | | | | | | | | | | | | | | |
| *Albizia kalkora* | ak | 75 | 7.71±0.43 | 2.00 | 19.0 | 1.01 | 0.89 | 0.48 | 4.41±0.24 | 1.56 | 9.81 | 8.25 | 0.74 | -0.46 | 159±13.3 | 89.1±38.7 | 1.10±0.14 | 47.3±1.88 |
| *Acer mandshuricum* | am | 75 | 4.65±0.30 | 2.00 | 22.0 | 4.18 | 24.38 | 0.56 | 3.50±0.20 | 1.17 | 11.6 | 10.4 | 2.08 | 6.49 | 57.0±3.66 | 43.5±5.58 | 1.19±0.04 | 47.1±0.25 |
| *Acer mono* | ao | 75 | 9.47±0.44 | 3.00 | 22.0 | 0.78 | 0.32 | 0.41 | 6.29±0.28 | 2.00 | 13.5 | 11.5 | 0.68 | 0.08 | 113±6.65 | 58.3±3.41 | 1.75±0.05 | 46.9±0.04 |
| *Acer tegmentosum* | at | 75 | 4.23±0.24 | 1.00 | 11.0 | 1.13 | 1.29 | 0.49 | 4.41±0.20 | 0.55 | 9.12 | 8.57 | 0.52 | 0.06 | 75.6±13.5 | 51.9±12.2 | 1.87±0.07 | 45.5±0.10 |
| *Corylus mandshurica* | cm | 75 | 7.03±0.32 | 3.00 | 18.0 | 1.30 | 2.50 | 0.39 | 5.67±0.36 | 1.48 | 16.7 | 15.3 | 1.36 | 1.80 | 146±50.0 | 130±20.1 | 1.71±0.04 | 45.8±0.19 |
| *Eleutherococcus senticosus* | es | 75 | 8.27±0.49 | 3.00 | 26.0 | 1.81 | 4.03 | 0.52 | 3.91±0.18 | 1.58 | 8.84 | 7.25 | 1.15 | 1.29 | 406±13.2 | 103±5.42 | 1.71±0.07 | 46.0±0.62 |
| *Fraxinus mandschurica* | fm | 73 | 7.38±0.49 | 3.00 | 21.0 | 1.36 | 1.29 | 0.57 | 3.46±0.15 | 1.56 | 8.83 | 7.27 | 1.30 | 3.30 | 114±7.84 | 50.8±3.33 | 2.32±0.06 | 46.8±0.14 |
| *Juglans mandshurica* | jm | 75 | 6.48±0.46 | 2.00 | 21.0 | 1.50 | 2.06 | 0.61 | 3.12±0.21 | 0.96 | 9.51 | 8.56 | 1.49 | 2.04 | 113±8.70 | 75.3±7.81 | 2.32±0.05 | 47.8±0.11 |
| *Larix gmelinii* | lg | 75 | 7.56±0.32 | 3.00 | 18.0 | 1.15 | 1.67 | 0.37 | 5.92±0.23 | 1.94 | 10.1 | 8.15 | 0.24 | -0.77 | 96.3±5.01 | 43.2±2.44 | 1.46±0.05 | 47.9±0.17 |
| *Phellodendron amurense* | pa | 75 | 3.49±0.14 | 2.00 | 9.00 | 1.78 | 5.45 | 0.35 | 1.20±0.05 | 0.65 | 2.58 | 1.94 | 1.18 | 1.52 | 56.3±3.60 | 37.8±2.58 | 2.53±0.06 | 47.3±0.11 |
| *Pinus densiflora* | pd | 75 | 9.68±0.56 | 2.00 | 21.0 | 0.66 | -0.63 | 0.50 | 7.86±0.48 | 0.79 | 19.1 | 18.3 | 0.66 | 0.08 | 55.9±4.01 | 31.6±1.76 | 1.37±0.01 | 46.0±1.02 |
| *Pinus koraiensis* | pk | 75 | 6.01±0.38 | 2.00 | 23.0 | 2.04 | 7.74 | 0.55 | 3.07±0.14 | 1.20 | 7.09 | 5.88 | 0.88 | 0.56 | 76.8±43.0 | 19.3±0.79 | 1.15±0.01 | 46.1±0.30 |
| *Picea asperata* | pa | 75 | 6.05±0.37 | 2.00 | 18.0 | 1.34 | 2.17 | 0.52 | 3.48±0.15 | 0.77 | 8.25 | 7.48 | 0.69 | 0.99 | 40.5±1.35 | 28.1±0.92 | 1.41±0.01 | 46.4±0.43 |
| *Quercus mongolica* | qm | 75 | 6.89±0.33 | 3.00 | 16.0 | 0.76 | -0.02 | 0.41 | 6.01±0.30 | 2.12 | 14.9 | 12.7 | 0.84 | 0.61 | 115±7.76 | 60.4±5.10 | 1.38±0.03 | 46.6±0.98 |
| *Sorbaria sorbifolia* | ss | 76 | 10.7±0.39 | 5.00 | 20.0 | 0.73 | 0.04 | 0.32 | 5.60±0.20 | 2.36 | 11.1 | 8.73 | 0.84 | 1.19 | 302±32.7 | 122±19.6 | 1.77±0.16 | 45.8±0.61 |
| *Tilia amurensis* | ta | 75 | 5.96±0.40 | 2.00 | 20.0 | 1.97 | 4.72 | 0.58 | 4.07±0.29 | 1.30 | 13.1 | 11.8 | 1.67 | 2.67 | 109±16.3 | 45.1±6.45 | 1.58±0.14 | 46.0±0.40 |
| *Ulmus davidiana* | ud | 75 | 11.5±0.62 | 3.00 | 30.0 | 1.00 | 0.97 | 0.47 | 8.36±0.68 | 2.36 | 27.0 | 24.6 | 1.46 | 1.43 | 128±33.8 | 71.1±16.0 | 1.73±0.05 | 46.5±0.23 |
| Maoershan forest (MES) | | | | | | | | | | | | | | | | | | |
| *Amygdalus davidiana* | ad | 91 | 7.75±0.41 | 3.00 | 22.0 | 1.37 | 1.64 | 0.50 | 5.86±0.26 | 2.40 | 17.8 | 15.4 | 1.48 | 4.33 | 192±17.7 | 76.9±1.73 | 1.57±0.16 | 47.0±1.57 |
| *Acer ginnala* | ag | 77 | 10.4±0.47 | 4.00 | 27.0 | 1.16 | 1.75 | 0.40 | 6.77±0.33 | 2.48 | 15.7 | 13.2 | 1.18 | 1.01 | 238±13.4 | 127±7.93 | 1.89±0.01 | 46.9±0.78 |
| *Acer mono* | ao | 75 | 9.03±0.52 | 2.00 | 26.0 | 1.48 | 2.45 | 0.50 | 5.84±0.24 | 1.74 | 10.5 | 8.74 | 0.28 | -0.63 | 99.5±8.89 | 40.3±2.12 | 1.95±0.04 | 47.4±0.10 |
| *Acer tegmentosum* | at | 75 | 7.72±0.43 | 3.00 | 21.0 | 0.99 | 0.80 | 0.48 | 4.67±0.25 | 1.80 | 11.6 | 9.80 | 0.97 | 0.49 | 175±17.3 | 60.2±2.56 | 2.06±0.04 | 47.2±0.14 |
| *Corylus mandshurica* | cm | 75 | 7.27±0.40 | 2.00 | 16.0 | 0.93 | 0.16 | 0.47 | 8.18±0.35 | 3.65 | 20.1 | 16.5 | 1.11 | 1.93 | 245±27.5 | 116±9.90 | 2.01±0.09 | 44.7±0.55 |
| *Eleutherococcus senticosus* | es | 73 | 7.68±0.31 | 2.00 | 14.0 | 0.52 | 0.12 | 0.35 | 4.70±0.25 | 1.71 | 15.4 | 13.7 | 2.01 | 6.81 | 529±55.9 | 109±7.86 | 1.80±0.07 | 47.0±0.08 |
| *Fraxinus mandschurica* | fm | 75 | 5.79±0.35 | 2.00 | 18.0 | 1.52 | 2.92 | 0.52 | 3.02±0.17 | 0.84 | 10.4 | 9.51 | 1.72 | 6.11 | 105±5.10 | 57.8±6.92 | 2.50±0.01 | 48.1±0.61 |
| *Juglans mandshurica* | jm | 76 | 9.33±0.53 | 2.00 | 21.0 | 0.77 | -0.27 | 0.49 | 4.75±0.20 | 1.47 | 9.66 | 8.19 | 0.67 | 0.46 | 195±19.9 | 108±3.88 | 2.75±0.04 | 48.8±0.48 |
| *Larix gmelinii* | lg | 77 | 6.55±0.36 | 2.00 | 18.0 | 1.23 | 1.83 | 0.49 | 4.05±0.14 | 1.94 | 7.03 | 5.08 | 0.34 | -0.63 | 46.7±3.87 | 31.6±2.57 | 1.80±0.01 | 47.8±0.12 |
| *Malus baccata* | mb | 75 | 6.68±0.37 | 3.00 | 17.0 | 1.42 | 1.41 | 0.48 | 3.89±0.21 | 1.07 | 9.60 | 8.53 | 1.44 | 1.96 | 319±50.9 | 153±17.2 | 1.76±0.06 | 44.4±0.99 |
| *Phellodendron amurense* | pa | 73 | 4.37±0.27 | 1.00 | 14.0 | 1.84 | 5.02 | 0.52 | 1.40±0.06 | 0.46 | 2.74 | 2.28 | 0.45 | 0.14 | 62.8±4.43 | 34.7±2.09 | 3.14±0.25 | 47.0±0.26 |
| *Padus racemose* | pr | 76 | 7.03±0.45 | 2.00 | 21.0 | 1.08 | 0.92 | 0.56 | 3.31±0.23 | 0.73 | 10.1 | 9.35 | 1.61 | 2.80 | 277±18.0 | 72.0±1.47 | 2.17±0.08 | 48.8±0.12 |
| *Syringa reticulate* | sr | 76 | 7.34±0.61 | 1.00 | 27.0 | 1.80 | 3.08 | 0.72 | 3.13±0.17 | 0.73 | 8.03 | 7.30 | 1.26 | 1.68 | 150±18.0 | 74.6±7.13 | 2.65±0.12 | 47.0±0.83 |
| *Sorbaria sorbifolia* | ss | 74 | 9.80±0.51 | 3.00 | 25.0 | 1.11 | 1.18 | 0.45 | 7.69±0.63 | 1.67 | 35.3 | 33.6 | 3.59 | 15.62 | 332±30.8 | 109±10.6 | 1.71±0.14 | 47.0±0.20 |
| *Ulmus davidiana* | ud | 88 | 12.03±0.6 | 4.00 | 33.0 | 1.05 | 1.05 | 0.47 | 6.90±0.26 | 2.99 | 18.5 | 15.5 | 1.33 | 4.16 | 361±22.3 | 117±15.8 | 2.12±0.01 | 46.4±0.55 |

**Supplementary Table 3.** continued

| Species | Abbr | No | BR | Mi_br_ | Ma_br_ | S_br_ | K_br_ | CV_br_ | BI | Mi_bi_ | Ma_bi_ | S_bi_ | K_bi_ | CV_bi_ | FSRL | SSRL | FN | FC |
| --- | --- | --- | --- | --- | --- | --- | --- | --- | --- | --- | --- | --- | --- | --- | --- | --- | --- | --- |
| Xianrendong forest (XRD) |  |  |  |  |  |  |  |  |  |  |  |  |  |  |  |  |  |  |
| *Actinidia argute* | aa | 76 | 7.00±0.42 | 2.00 | 16.0 | 0.80 | -0.15 | 0.53 | 3.16±0.20 | 0.70 | 8.41 | 7.71 | 1.24 | 0.90 | 109±11.0 | 41.1±2.56 | 2.71±0.23 | 45.7±0.18 |
| *Acer ginnala* | ag | 75 | 8.60±0.52 | 2.00 | 31.0 | 2.12 | 7.12 | 0.52 | 4.85±0.23 | 0.95 | 11.9 | 10.9 | 0.88 | 1.25 | 276±8.77 | 122±11.3 | 1.79±0.02 | 46.2±1.00 |
| *Alnus japonica* | aj | 75 | 9.24±0.60 | 2.00 | 23.0 | 0.60 | -0.65 | 0.56 | 4.23±0.26 | 0.99 | 10.0 | 9.01 | 0.59 | -0.55 | 176±91. 9 | 23.3±5.12 | 2.88±0.28 | 48.0±0.22 |
| *Albizia kalkora* | ak | 75 | 7.25±0.38 | 2.00 | 17.0 | 0.70 | -0.25 | 0.45 | 4.10±0.21 | 1.82 | 10.7 | 8.89 | 1.32 | 1.77 | 226±21.4 | 95.3±7.31 | 1.99±0.03 | 47.8±0.12 |
| *Fraxinus mandschurica* | fm | 74 | 3.89±0.23 | 1.00 | 10.0 | 1.15 | 1.06 | 0.52 | 1.31±0.07 | 0.29 | 3.00 | 2.71 | 0.87 | 0.46 | 35.9±4.29 | 25.7±2.38 | 1.76±0.09 | 46.0±0.54 |
| *Juglans mandshurica* | jm | 75 | 8.07±0.43 | 2.00 | 23.0 | 1.06 | 1.95 | 0.46 | 4.67±0.42 | 1.66 | 30.8 | 29.2 | 5.25 | 34.1 | 88.3±7.34 | 41.8±3.71 | 2.31±0.15 | 48.1±0.01 |
| *Larix gmelinii* | lg | 75 | 6.25±0.34 | 2.00 | 15.0 | 0.87 | 0.31 | 0.48 | 3.65±0.16 | 1.58 | 8.56 | 6.98 | 0.85 | 1.01 | 104.±15.9 | 71.0±7.77 | 1.74±0.02 | 44.2±0.42 |
| *Lindera obtusiloba* | lo | 75 | 4.71±0.28 | 2.00 | 13.0 | 1.54 | 2.47 | 0.52 | 1.50±0.06 | 0.61 | 3.36 | 2.75 | 0.92 | 1.37 | 102±24.0 | 64.6±11.8 | 3.12±0.75 | 46.8±1.59 |
| *Magnolia sieboldii;* | ms | 75 | 3.75±0.23 | 2.00 | 16.0 | 3.25 | 15.70 | 0.54 | 1.39±0.05 | 0.65 | 2.62 | 1.97 | 0.60 | -0.24 | 61.7±4.99 | 41.2±2.79 | 1.70±0.05 | 48.3±0.54 |
| *Phellodendron amurense* | pa | 75 | 4.63±0.27 | 2.00 | 15.0 | 1.75 | 4.07 | 0.51 | 1.59±0.07 | 0.56 | 3.10 | 2.53 | 0.76 | -0.44 | 76.2±6.82 | 38.9±4.46 | 2.58±0.13 | 46.7±0.71 |
| *Pinus densiflora* | pd | 75 | 4.37±0.21 | 2.00 | 13.0 | 1.79 | 5.16 | 0.43 | 5.09±0.24 | 2.10 | 13.7 | 11.6 | 1.34 | 2.60 | 85.5±3.25 | 45.0±2.32 | 1.24±0.20 | 46.0±0.81 |
| *Pinus koraiensis* | pk | 75 | 4.79±0.23 | 2.00 | 12.0 | 1.31 | 1.91 | 0.41 | 3.83±0.18 | 1.69 | 10.5 | 8.85 | 1.70 | 4.10 | 42.4±3.53 | 24.6±2.91 | 1.31±0.01 | 44.2±0.37 |
| *Picea wilsonii* | pw | 73 | 6.89±0.42 | 2.00 | 19.0 | 1.42 | 2.22 | 0.52 | 3.67±0.19 | 1.30 | 8.53 | 7.23 | 1.28 | 1.29 | 57.0±8.13 | 33.2±2.62 | 1.84±0.07 | 45.6±0.64 |
| *Quercus acutissima* | qa | 77 | 8.17±0.35 | 3.00 | 18.0 | 1.04 | 1.04 | 0.37 | 8.09±0.35 | 3.29 | 16.7 | 13.5 | 0.73 | 0.17 | 124±22.7 | 51.0±5.87 | 1.71±0.04 | 43.6±0.66 |
| *Quercus mongolica* | qm | 75 | 7.67±0.43 | 3.00 | 26.0 | 1.96 | 6.20 | 0.49 | 8.22±0.49 | 2.59 | 23.4 | 20.8 | 1.42 | 2.24 | 151±16.3 | 74.0±6.18 | 1.64±0.11 | 45.8±0.21 |
| *Rhus chinensis* | rc | 75 | 7.05±0.36 | 2.00 | 17.0 | 0.73 | -0.20 | 0.44 | 4.12±0.19 | 1.40 | 8.33 | 6.93 | 0.37 | -0.46 | 222±25.6 | 79.8±10.1 | 1.61±0.01 | 47.3±0.38 |
| *Symplocos paniculata* | sp | 75 | 4.99±0.32 | 1.00 | 19.0 | 2.11 | 7.26 | 0.56 | 1.88±0.11 | 0.30 | 4.35 | 4.05 | 0.79 | 0.11 | 189±22.4 | 86.2±4.42 | 2.43±0.09 | 46.5±0.52 |
| *Ulmus laciniata* | ul | 75 | 9.47±0.53 | 2.00 | 25.0 | 0.96 | 1.24 | 0.49 | 9.28±0.45 | 2.19 | 24.4 | 22.2 | 0.89 | 1.88 | 171±30.8 | 49.8±7.46 | 1.81±0.15 | 46.4±0.27 |
| *Zanthoxylum simulans* | zs | 75 | 5.11±0.27 | 2.00 | 12.0 | 1.34 | 1.15 | 0.46 | 1.49±0.07 | 0.40 | 3.83 | 3.44 | 1.29 | 2.06 | 99.2±13.7 | 50.2±3.80 | 2.83±0.01 | 49.3±0.39 |

Abbr, abbreviation of species; No, number of individual second-order fine roots; Mi_br_, minimum value of BR; Ma_br_, maximum value of BR; S_br_, skewness of BR; K_br_, kurtosis of BR; CV_br_, coefficient of variation of BR; Mi_bi_, minimum value of BI; Ma_bi_, maximum value of BI; S_bi_, skewness of BI; K_bi_, kurtosis of BI; CV_bi_, coefficient of variation of BI.

**Supplementary Table 4.** Relationships of first-order fine-root length (FL), diameter (FD) and area (FA) with branching intensity (BI) and ratio (BR) for Changbai Mountain (CBS), Maoershan (MES) and Xianrendong (XRD) forests, as estimated from linear mixed-effects kinship models controlling for phylogeny with a tree species level of phylogeny and including tree species as a random effect. Data were log-transformed before analyses. Estimates are given with their standard error (SE), along with the z-value and *p*-value from the model.

| Response variable | | FL |  |  |  | FD |  |  |  | FA |  |  |  |
| --- | --- | --- | --- | --- | --- | --- | --- | --- | --- | --- | --- | --- | --- |
| Explanatory variable | | Estimate | SE | z-value | *p*-value | Estimate | SE | z-value | *p*-value | Estimate | SE | z-value | *p*-value |
| CBS | Intercept | 2.5493 | 0.1423 | 17.9 | <0.001 | -1.1277 | 0.3332 | -3.38 | <0.001 | 2.5500 | 0.3167 | 8.05 | <0.001 |
|  | Log(BI) | -0.8851 | 0.0929 | -9.53 | <0.001 | -0.9735 | 0.2176 | -4.47 | <0.001 | -1.7852 | 0.2068 | -8.63 | <0.001 |
|  | Intercept | 3.3074 | 0.6374 | 5.19 | <0.001 | 0.9010 | 0.6337 | 1.42 | 0.160 | 5.2971 | 0.9462 | 5.60 | <0.001 |
|  | Log(BR) | -1.0374 | 0.3196 | -3.25 | <0.01 | -1.7464 | 0.3178 | -5.49 | <0.001 | -2.7094 | 0.4745 | -5.71 | <0.001 |
| MES | Intercept | 2.7514 | 0.2098 | 13.1 | <0.001 | -1.1627 | 0.4495 | -2.59 | <0.01 | 2.7363 | 0.4894 | 5.59 | <0.001 |
|  | Log(BI) | -0.9423 | 0.1370 | -6.88 | <0.001 | -0.7348 | 0.2936 | -2.50 | <0.05 | -1.6621 | 0.3197 | -5.20 | <0.001 |
|  | Intercept | 3.8134 | 0.9256 | 4.12 | <0.001 | -0.2551 | 1.2609 | -0.20 | 0.840 | 4.6676 | 1.8066 | 2.58 | <0.01 |
|  | Log(BR) | -1.2161 | 0.4588 | -2.65 | <0.01 | -0.9880 | 0.6250 | -1.58 | 0.110 | -2.1741 | 0.8955 | -2.43 | <0.05 |
| XRD | Intercept | 2.6034 | 0.0726 | 35.9 | <0.001 | -1.3210 | 0.1151 | -11.5 | <0.001 | 2.5025 | 0.2438 | 10.3 | <0.001 |
|  | Log(BI) | -0.9261 | 0.0536 | -17.3 | <0.001 | -0.5797 | 0.0850 | -6.82 | <0.001 | -1.4389 | 0.1800 | -7.99 | <0.001 |
|  | Intercept | 3.9315 | 0.6860 | 5.73 | <0.001 | -0.1782 | 0.4649 | -0.38 | 0.700 | 4.6706 | 1.2180 | 3.83 | <0.001 |
|  | Log(BR) | -1.3594 | 0.3781 | -3.60 | <0.001 | -1.0249 | 0.2562 | -4.00 | <0.001 | -2.1707 | 0.6714 | -3.23 | <0.001 |
| All data | Intercept | 2.5390 | 0.1593 | 15.9 | <0.001 | -1.2339 | 0.1617 | -7.63 | <0.001 | 2.5755 | 0.1996 | 12.9 | <0.001 |
|  | Log(BI) | -0.8361 | 0.0494 | -16.9 | <0.001 | -0.7496 | 0.1104 | -6.79 | <0.001 | -1.6217 | 0.1363 | -11.9 | <0.001 |
|  | Intercept | 3.1992 | 0.4918 | 6.50 | <0.001 | 0.2713 | 0.4331 | 0.63 | 0.530 | 4.6964 | 0.9130 | 5.14 | <0.001 |
|  | Log(BR) | -0.9677 | 0.1959 | -4.94 | <0.001 | -1.3196 | 0.2242 | -5.89 | <0.001 | -2.2721 | 0.3527 | -6.44 | <0.001 |

**Supplementary Table 5.** The proportion of variation explained (top panel) and loading scores (bottom panel) for each component from principal component analysis on the fine-root traits and their inequality/variation. Abbreviations are given in Figure 4.

| Component | Eigenvalue | Proportion | Cumulative |
| --- | --- | --- | --- |
| 1 | 7.65 | 42.5 | 42.5 |
| 2 | 2.77 | 15.4 | 57.9 |
| 3 | 2.34 | 13.0 | 70.9 |
| 4 | 1.15 | 6.38 | 77.3 |
| 5 | 0.96 | 5.34 | 82.6 |
| 6 | 0.94 | 5.22 | 87.8 |
| 7 | 0.59 | 3.29 | 91.1 |
| 8 | 0.46 | 2.57 | 93.7 |
| Variable | Component 1 | Component 2 | Component 3 |
| FL | 0.33 |  | 0.19 |
| FD | 0.33 |  |  |
| FA | 0.32 | 0.12 |  |
| FSRL | -0.12 | -0.40 | 0.20 |
| SL | 0.30 |  | 0.31 |
| SD | 0.33 |  |  |
| SA | 0.34 |  | 0.12 |
| SSRL | -0.16 | -0.35 | 0.26 |
| BR | -0.24 | -0.20 | 0.16 |
| BI | -0.31 |  |  |
| FN | 0.20 | -0.12 | 0.25 |
| FC | 0.13 | -0.13 | 0.25 |
| G_fa_ | -0.14 | 0.41 | 0.14 |
| G_fl_ |  | 0.11 | 0.56 |
| G_fd_ | -0.17 | 0.46 |  |
| CV_fl_ | -0.14 | 0.16 | 0.47 |
| CV_fd_ | -0.19 | 0.36 |  |
| CV_fa_ |  | 0.27 |  |
